# Altered steady state and activity-dependent de novo protein expression in fragile X syndrome

**DOI:** 10.1038/s41467-019-09553-8

**Published:** 2019-04-12

**Authors:** Heather Bowling, Aditi Bhattacharya, Guoan Zhang, Danyal Alam, Joseph Z. Lebowitz, Nathaniel Bohm-Levine, Derek Lin, Priyangvada Singha, Maggie Mamcarz, Rosemary Puckett, Lili Zhou, Sameer Aryal, Kevin Sharp, Kent Kirshenbaum, Elizabeth Berry-Kravis, Thomas A. Neubert, Eric Klann

**Affiliations:** 1 Center for Neural Science, New York University, New York, NY 10003 USA; 2 Centre for Brain Development and Repair, Institute for Stem Cell Biology and Regenerative Medicine, Bangalore, 560065 India; 3 Skirball Institute for Biomolecular Medicine and Department of Cell Biology, New York University School of Medicine, New York, NY 10016 USA; 4 Department of Pediatrics, Rush University Medical Center, Chicago, IL 60612 USA; 5 Molecular Pharmacology Program, Sackler Institute of Graduate Biomedical Sciences, New York University School of Medicine, New York, NY 10016 USA; 6 Department of Chemistry, New York University, New York, NY 10003 USA; 7 Department of Neurological Sciences and Biochemistry, Rush University Medical Center, Chicago, IL 60612 USA; 8 NYU Neuroscience Institute, New York University School of Medicine, New York, NY 10016 USA

## Abstract

Whether fragile X mental retardation protein (FMRP) target mRNAs and neuronal activity contributing to elevated basal neuronal protein synthesis in fragile X syndrome (FXS) is unclear. Our proteomic experiments reveal that the de novo translational profile in FXS model mice is altered at steady state and in response to metabotropic glutamate receptor (mGluR) stimulation, but the proteins expressed differ under these conditions. Several altered proteins, including Hexokinase 1 and Ras, also are expressed in the blood of FXS model mice and pharmacological treatments previously reported to ameliorate phenotypes modify their abundance in blood. In addition, plasma levels of Hexokinase 1 and Ras differ between FXS patients and healthy volunteers. Our data suggest that brain-based de novo proteomics in FXS model mice can be used to find altered expression of proteins in blood that could serve as disease-state biomarkers in individuals with FXS.

## Abstract (web summary)

Elevated protein synthesis, and dysregulated mGluR signalling, are documented in fragile X syndrome (FXS) Here the authors use proteomic analysis in a mouse model of FXS, and following mGluR5 stimulation, to identify potential biomarkers for the disease.

## Introduction

Fragile X syndrome (FXS) is caused by a trinucleotide repeat expansion in the 5′ promoter region of the *FMR1* gene, leading to transcriptional silencing and loss of its protein product fragile X mental retardation protein (FMRP). Loss of FMRP in FXS has been shown to cause exaggerated translation in multiple brain areas in FXS model mice, flies, and patient-derived fibroblasts^1–4^. In most cases a 5–20% increase in steady state/basal translation has been reported in FXS models, with only a minimal increase upon stimulation of cell surface receptors such as metabotropic glutamate receptor 5 (mGluR5; ref. ^5^). Proteomic studies in FXS model mice have been performed in cell cultures, and SILAC-labeled and iTRAQ-labeled cortical synaptoneurosomes, but they were limited to only the developing or the synaptic proteome^6–8^. In parallel, basic mechanistic studies have offered valuable insights into FMRP action by focusing on single candidate mRNAs such as Arc/Arg 3.1, PSD-95, and MAP1B^9–11^. These studies have advanced the basic biology of FMRP function; however, there is an acute need for unbiased protein biomarkers for FXS to identify patient subgroups and treatment response. This lack of unbiased biomarkers has contributed to the failure of multiple clinical trials recently^12–14^. Therefore, it is critical to develop approaches for unbiased patient monitoring of treatment efficacy and to identify, catalog, and track patient subgroups for the best possible clinical trial outcomes^15^. To date, no studies have attempted to identify proteomic candidates from mass spectrometric (MS) screens that can translate into usable biomarkers.

This gap in candidate-based studies and solution-driven approaches arises from the fact that little is understood about the specific transcripts that are translated inappropriately in FXS. The current key questions in the field are: (1) What proteins change in the brain that underlie FXS pathology regardless of their localization or purported function? (2) Are disruptions in protein synthesis the result of an inappropriate response to activity or do they occur at the homeostatic level? (3) Are these candidates sensitive to drug treatments that have been shown to rescue phenotypes in FXS model mice and humans? (4) Do the same proteins show dysregulation in more accessible tissues for biomarker discovery?

We began to address these questions by comparing steady state differences in translation using a quantitative de novo protein synthesis-labeling technique in acutely prepared hippocampal slices from wild-type and FXS model mice. BONLAC is a combinatorial proteomic profiling technique^16–18^ that uses the click-chemistry biorthogonal noncanonical amino acid tagging (BONCAT) technique to identify de novo peptides and stable isotopic labeling by amino acids in cell culture (SILAC) proteomics to measure protein abundance^16,19,20^. Because multiple studies have shown that altered mGluR5 signaling plays an important role in FXS pathophysiology^21^, we additionally carried out BONLAC to identify proteins synthesized in response to stimulation of mGluR5 signaling in both wild-type and FXS model mice. Next, we identified candidates from our two BONLAC screens, and compared them with previously published lists of FMRP targets and ASD-susceptibility genes, and validated by western blotting the de novo expression levels of the most promising target proteins. We evaluated the cross-tissue applicability of the candidates by testing the whole blood response of FXS model mice to treatment with pharmacological agents that ameliorate pathophysiology and their abundance in plasma from FXS individuals.

## Results

Previous publications monitoring new protein synthesis have reported a net increase in S^35^, puromycin, or azido-homoalanine (AHA) incorporation, suggesting elevated de novo translation in *Fmr1* knockout (KO, FXS model) mice^5,22,23^. However, specific changes in de novo translation were not assessed in these studies due to technical limitations in measuring the proteome. Several studies have attempted to circumvent these limitations by identifying FMRP-associated mRNAs^24^, or quantifying individual proteins of interest by measuring their relative abundance with western blotting. To date, there has been no large unbiased screen of de novo protein synthesis in the mature brain of FXS model mice to establish which transcripts are differentially synthesized versus those that accumulate over time in FXS model mice.

**Fig. 1:**
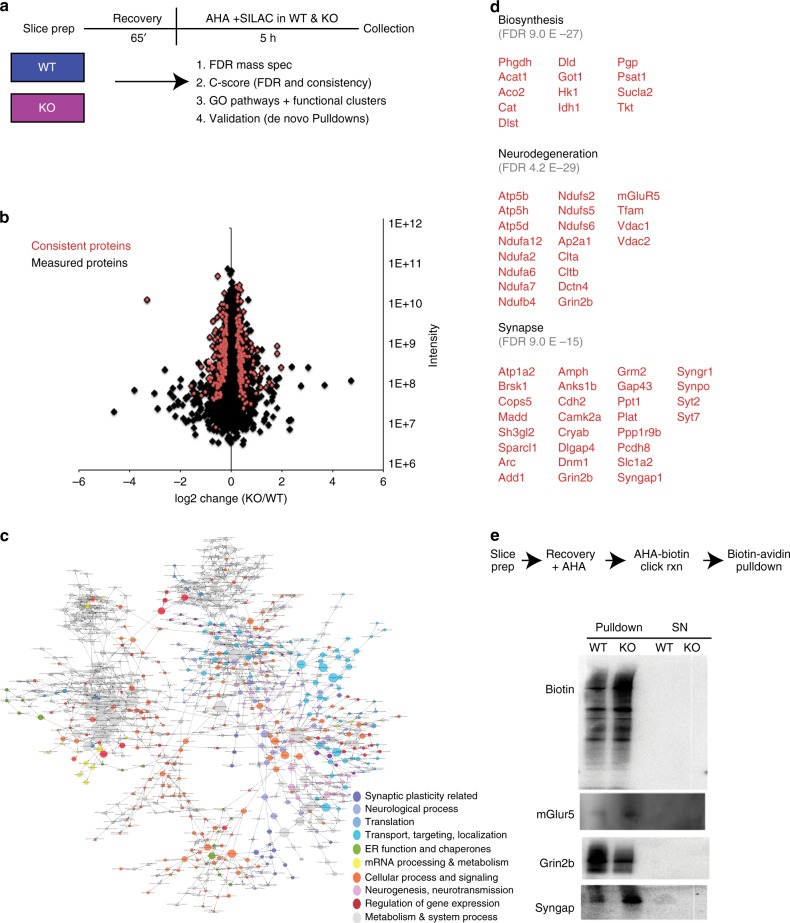
Steady state de novo proteome in the hippocampus of wild-type and *Fmr1* knockout mice. **a** Schematic timeline and workflow of the BONLAC experiment in hippocampal slices from wild-type (WT) and *Fmr1* knockout (KO) mice. Data are from 3 biological replicate experiments (*n* = 3 mice/treatment/experiment for 3 separate experiments, therefore *n* = 9/genotype split pooled into groups of 3). **b** Fold change vs intensity distribution plot of all detected proteins in KO versus WT hippocampal proteome in black. Overlay with red scatter plot depicts candidate proteins that were consistently altered using cut-offs derived from C-score rank score algorithm. **c** BinGO Cytoscape depiction of enriched gene ontology groups. **d** Top functional clusters in *Fmr1* KO mice compared to WT littermates using DAVID 6.8. **e** AHA-pull down validations (*n* = 3 mice per genotype in WT/KO sets)

We performed BONLAC proteomics on acutely prepared dorsal hippocampal slices from 10–12 week old FXS model mice and their wild-type (WT) littermates, and measured polypeptide accumulation over 5 h of labeling (Fig. 1a). Ten to twelve week old mice were selected as this age best matches studies that have reported changes in FXS-like behavior^22,23,25^. We performed BONLAC three separate times using three different sets of litter and age-matched mice for a total of three independent biological replicates with three animals pooled in each treatment group for each study (*n* = 9 WT, *n* = 9 KO pooled in 3 groups of 3 mice per pool). In addition, we alternated SILAC labeling (medium or heavy) between each of the three biological replicates to control for any potential labeling bias. Proteins of interest or “candidates” from this BONLAC screen were identified using a multi-tiered statistical analysis based on coincidence detection. Candidates were required to: (1) meet standard mass spectrometry identification Benjamini–Hochberg FDRs < 0.05 at the protein level (Supplementary Data 1), (2) meet Perseus FDR criteria for intensity, and 3) the directional change such that differences in protein amount had to be significant with *p* < 0.3, had to be measured at least twice, and consistently changed in the same direction every time measured (“C-score”, Fig. 1b, Supplementary Data 1, method previously established in ref. ^16^). When taken alone these criteria for selecting candidates for differential regulation were not statistically stringent enough to ensure high confidence. Therefore, proteins that met these criteria then were subjected to gene ontology (GO) analysis (Supplementary Data 2), and only those identified as significantly enriched (*p* < 1E−5 after FDR correction) were considered for further independent verification by western blotting. The level of dual AHA and SILAC signal in all peptides measured was present as reported previously^16,17^ ruling out any inefficiencies in labeling.

In all, 2641 proteins were measured across all three biological replicates and 324 consistently altered across three biological replicates (Supplementary Data 1 and Fig. 1b). At steady state, we found that the expression of some of the newly synthesized proteins were upregulated and some were downregulated in the hippocampus of *Fmr1* KO mice compared to wild-type, with higher-fold changes being recorded for upregulated proteins. GO analysis using BiNGO and DAVID 6.8 indicated that the top enriched functional clusters were biosynthesis, protein folding, synaptic proteins, and neurodegeneration-related proteins (including metabolism targets) (Fig. 1c, d, Supplementary Data 2). These findings suggest that the whole circuit protein synthesis includes both synaptic- and metabolism-specific responses, the latter being poorly studied in FXS.

We then validated candidates using BONCAT-specific pull-downs (Fig. 1e) to confirm the predictive power of the technique. Hippocampal slices were labeled with AHA and then lysed, subjected to cycloaddition conjugation, and the resultant AHA-biotin tagged proteins (newly synthesized proteins) were isolated with avidin beads (*n* = 3 biological replicates per protein). Newly synthesized proteins then were run on western blots and individual candidates from the mass spectrometry list were probed (Fig. 1e). We first confirmed that pull-down of only newly synthesized proteins showed an upregulation of protein synthesis in *Fmr1* KO hippocampal slices (“biotin” top panel), then probed for three candidates that met the cutoff, consistency, and GO criteria: mGluR5, NMDAR2B/Grin2B/GluN2B/NR2B2b (Grin2b) and SynGAP, all of which changed according to the prediction by mass spectrometry (up, down, and up respectively in the *Fmr1* KO mice) (Fig. 1e). These data confirm that BONLAC predicts measurable de novo changes in the proteome.

**Fig. 2:**
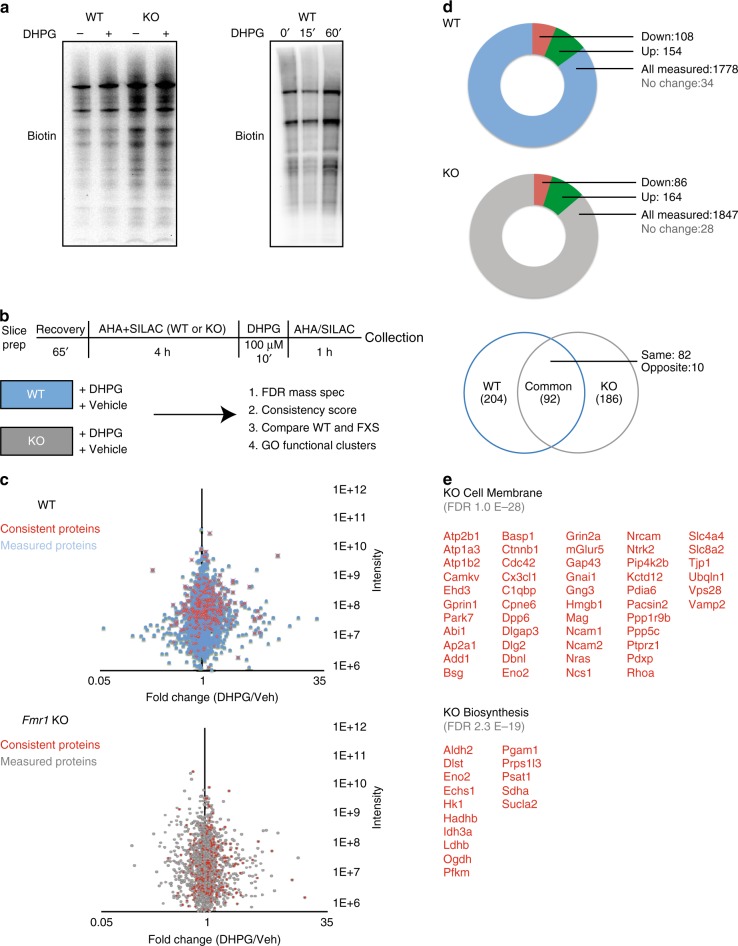
mGluR-stimulated de novo proteome differs in the hippocampus of wild-type and *Fmr1* knockout mice. **a** Example western blot image of BONCAT labeling of biotinylated de novo proteins made in wild-type (WT) and *Fmr1* knockout (KO) hippocampal slices in response to the group I mGluR agonist DHPG (left panel, *n* = 3 WT with slices split evenly between Veh and DHPG, *n* = 3 KO with slices split evenly between treatments, *p* = 0.04; WT Veh vs WT DHPG, *p* = 0.06 WT Veh vs KO Veh, *p* = 0.32 KO Veh vs KO DHPG). In keeping with previous reports, no appreciable change in labeling was observed seen in KO slices stimulated with DHPG. (right panel). Sample western blot of time course of DHPG-induced de novo protein synthesis (right panel, *n* = 3 0′ and 60′, *p* = 0.05, Student’s *t*-test, *n* = 2 for 15′ timepoint). **b** Schematic timeline and workflow of the BONLAC experiment. **c** Screens were performed for WT and KO separately. Both followed the same workflow and processing and were done on successive days from same batch of litters. Fold change vs intensity distribution plot of all detected proteins in WT +/− DHPG (top) or KO +/− (bottom) hippocampal proteome in blue and gray respectively (*n* = 9 WT Veh, *n* = 9 WT DHPG, *n* = 8 KO Veh, *n* = 8 KO DHPG divided in 3 biological replicate groups per treatment condition per genotype). Overlay with red scatter plot depicts candidate proteins that were consistently altered using cut-offs derived from C-score rank score algorithm. *n* = 3 MS runs for 3 mice per genotype, per condition, per run. **d** Pie-chart breakup of total number of proteins measured across all runs and consistently upregulated and downregulated candidates from WT and KO screens (top and middle panel). Venn diagram depicting overlap in candidate identities between WT + DHPG and KO + DHPG screens (bottom panel). **e** Top Gene Ontology candidates in KO DHPG screen in Cell Membrane and Biosynthesis categories from DAVID 6.8

Protein synthesis dysregulation downstream of mGluR5 has been implicated in FXS pathology^26^. Previous reports have demonstrated that the mGluR1/5 agonist, DHPG, induces increased protein synthesis in wild-type, but not *Fmr1* KO mice, further suggesting a disorganized proteomic response to glutamatergic activity^5^. We first confirmed that DHPG-induced protein synthesis was disrupted in FXS model mice compared to their wild-type littermates (Fig. 2a left panel, quantification in Supplementary Fig. 1) and that we were using a temporal window that provided optimal protein synthesis recovery (Fig. 2a right panel, quantification in Supplementary Fig. 1). Then we performed BONLAC on acute adult hippocampal slices and compared the response between wild-type and *Fmr1* KO littermates with and without DHPG (Fig. 2b, *n* = 9 WT, *n* = 8 KO/group, pooled into groups of 3 in 3 biological replicates/group).

We analyzed which proteins were consistently altered in wild-type and *Fmr1* KO responses to DHPG across runs (2/2 or 3/3 had to change in the same direction). Consistent proteins were called “candidates” (red proteins vs blue or gray in Fig. 2c, d). 1778 proteins were identified across three biological replicates for wild-type slices + DHPG, with a total of 296 hits consistently changed across two replicates or more (Supplementary Data 3). Of these, 154 were upregulated by at least 10% and 108 downregulated by at least 10% (Fig. 2d). In *Fmr1* KO slices treated with DHPG, we identified 1847 proteins, with 278 that consistently changed (Supplementary Data 3). Of these, 164 were upregulated, whereas 86 were downregulated by at least 10% (Fig. 2d). These results suggest that more proteins are measured but fewer are consistent across runs in FXS model mice (Fig. 2d and Supplementary Data 2). We compared these candidate proteins that consistently changed in response to DHPG in the wild-type and *Fmr1* KO mice for overlap and noted that there were relatively fewer changes in common than we expected (Fig. 2d). Only 82 candidate proteins consistently changed in the same direction between wild-type and *Fmr1* KO slices stimulated with DHPG, suggesting the bulk of the consistently changed proteins were different between wild-type and FXS model mice (Fig. 2d). We analyzed the consistently changed proteins in each group with DAVID GO analysis (version 6.8), examining those proteins that were only changed in *Fmr1* KO mice (Fig. 2e), only changed in wild-type mice, consistently changed between genotype, those with degree of difference response, and those opposite responses using DAVID (Supplementary Data 4). Only those GO groups with a significant FDR were included in the narrowed list of candidates for validation (Fig. 2e, Supplementary Data 4). We then asked which types of proteins and pathways were most altered in *Fmr1* KO hippocampi following DHPG stimulation using a DAVID analysis (Supplementary Data 4) and found that two major categories exhibited a differential response: cell membrane and biosynthesis proteins (Ras from cell membrane and Hexokinase 1 (Hk1) from biosynthesis validated in Supplementary Fig. 2). Thus, we have identified different de novo proteins that are synthesized in response to DHPG in both wild-type and *Fmr1* KO hippocampi that have limited overlap, suggesting a different FXS proteomic response to mGluR stimulation that includes changes in cell membrane and biosynthesis proteins.

**Fig. 3:**
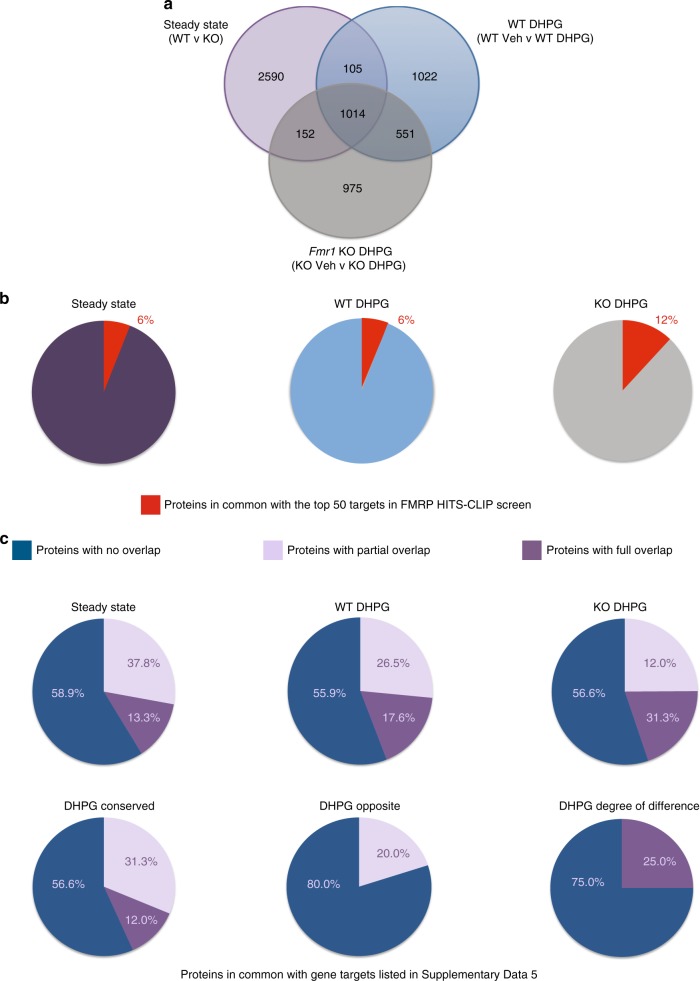
Steady state and mGluR-stimulated de novo proteomes differ in *Fmr1* knockout mice. **a** Venn diagram of proteins measured in steady state, wild-type (WT) DHPG and *Fmr1* knockout (KO) DHPG BONLAC experiments. We noted that despite many proteins measured in common, there is limited overlap in proteins being made, suggesting distinct de novo proteomic responses at steady state and in response to DHPG treatment. **b** Pie charts of the top 50 FMRP targets from HITS-CLIP^24^ and the consistently changed proteins in steady state, WT DHPG and FXS DHPG BONLAC studies. **c** Pie charts of top ASD genes and the BONLAC experiments (source of gene lists are in Supplementary Data 5)

The findings presented in Fig. 2c suggest that the mGluR-induced alterations in new protein expression in *Fmr1* KO and wild-type slices are different and use different proteins to mount their responses. We then compared the DHPG-induced proteins in wild-type and *Fmr1* KO slices to the steady state and found very limited overlap between the three groups, which was also the case in the consistent responders (Fig. 3a, Supplementary Fig. 3). To control for potential differences between MS experiments, we analyzed the total proteins measured in the steady state and DHPG stimulation experiments. The total proteins measured in each experiment did not differ greatly despite the candidate lists of newly synthesized proteins being different (Fig. 3a). The observation that there were few conserved proteins that consistently changed between wild-type and *Fmr1* KO slices after DHPG stimulation, and little overlap with the steady state differences reported earlier suggests that a different pool of proteins are being synthesized in FXS model mice under homeostatic conditions and following group I mGluR stimulation.

Given the previous hypothesis that FMRP mRNA targets would account for the bulk of proteins differentially synthesized in FXS model mice, we compared our consistently altered proteins with FMRP-binding mRNAs identified by HITS-CLIP that was performed in brains of *Fmr1* KO mice of a similar age^24^. Only a small number—6%—of our steady state candidates overlapped with the top 50 HITS-CLIP list (Fig. 3b, Supplementary Data 5). The overlap with FMRP target mRNA dataset^24^ did not drastically increase—6% with wild-type + DHPG only targets and 12% with *Fmr1* KO + DHPG targets (Fig. 3b). Therefore, despite the identification of some canonical FMRP targets by the BONLAC screens, the bulk of the proteins that change consistently were not previously identified as top FMRP mRNA targets, suggesting that the de novo proteome under both steady state and activity-dependent conditions is not chiefly comprised of FMRP mRNA targets in FXS model mice under our experimental conditions.

Because FXS is the leading monogenic cause of ASD, we also examined the relevance of the de novo protein candidate list to targets identified in the larger autism spectrum disorder (ASD) field. To determine whether the dataset was enriched for candidates associated with ASD, we first compiled a database of all genes/mRNA or protein targets from 22 different ASD databases comprising 1941 targets (Fig. 3c, Supplementary Data 5, tab “steady state”). Upon comparing the two sets, we found that of the 324 consistently changed steady state proteins in the FXS model mice in the BONLAC screen, 32 were represented in the ASD dataset (9.08%) with direct sequence identity and 57 targets overlapped as isoform variants (17.59% overlap) (Fig. 3b). Of these, maximal overlap was found in neuronal projection and synaptic plasticity groups.

We next cross-referenced the DHPG-stimulated de novo protein candidates in both genotypes to the ASD hits database (Fig. 3b, Supplementary Data 5, “DHPG” tabs). We found that 35/296 wild-type and 38/279 *Fmr1* KO protein candidates showed overlap with the ASD database, whereas 10/82 protein candidates conserved between the wild-type and *Fmr1* KO lists were ASD targets. To combat potential protein identification mismatches due to software differences and to include related proteins, we also included “partial hits” (Fig. 3b, Supplementary Data 5), which increased the overlap; however, the bulk of the candidates remain previously unreported in previous ASD publications. Comparison to a random database did not yield a high overlap of enriched proteins either despite both datasets measuring common proteins (Supplementary Fig. 4). These data are notable because multiple genes have been reported to be potential ASD risk factors, but their relevance to ASD pathology has only been shown in a small number of studies. In summary, our screen of steady state de novo translation in *Fmr1* KO mice revealed changes in proteins associated with synaptic function and metabolism that have little overlap with previously reported FMRP-binding mRNAs, with some commonalities corresponding to previously reported general ASD-associated proteins.

Because FMRP is an RNA-binding protein and affects multiple aspects of RNA biology, we queried whether there were any unifying properties of the mRNAs that correspond to the consistently altered proteins. Although there are some trends such as increased transcript length and changes in UTR lengths, overall there was no one unifying sequence that could be found in the mRNAs corresponding to proteins altered in the FXS model mice at steady state or in the DHPG-stimulated de novo proteomes in the wild-type or FXS model mice (Supplementary Fig. 5, Supplementary Methods). These data suggest that regulation of protein synthesis in the absence of FMRP is more complex than has previously been reported, and the loss of FMRP does not affect a single proteomic response, but both homeostatic and activity-dependent conditions.

Next we asked whether the validated BONLAC targets could be used to correlate with drug efficacy and/or also were dysregulated in human FXS patients. In addition, unlike genetically homogenous mice with littermates that have similar environmental conditions, patients are far more complex due to heterogeneity in FMRP expression, genetic and epigenetic backgrounds, environmental influences, behavioral phenotype, and response to drug treatments. Therefore, the ability to detect different molecular signatures in FXS patients that could both stratify them into subpopulations and potentially predict whether they are part of a subset that responds to a given drug would also be critical for future clinical trials for FXS. Because living human patients can only provide readily biopsiable tissues such as blood, skin, or urine and FMRP loss occurs throughout the body, examining the BONLAC targets we have identified in *Fmr1* KO mouse brains in an accessible tissue such as blood was a logical next step.

**Fig. 4:**
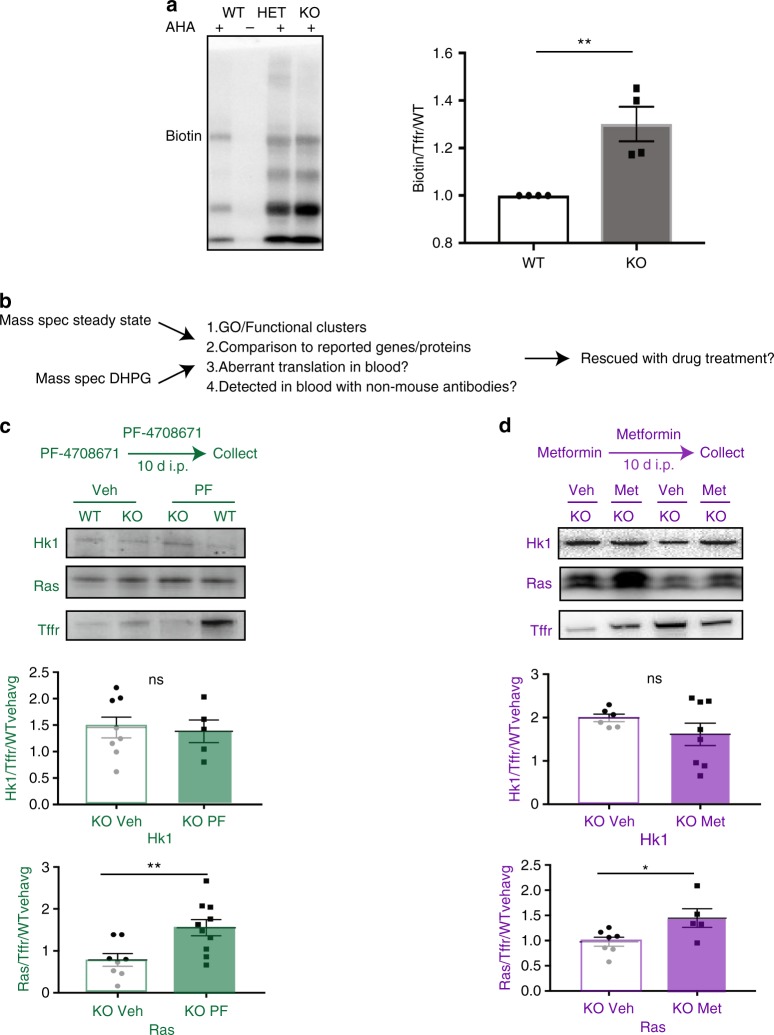
Whole blood levels of RAS and HK1 are altered in *Fmr1* knockout mice in a treatment-dependent manner. **a** De novo protein synthesis is altered in *Fmr1* knockout (KO) mouse whole blood compared to wild-type (*n* = 4 WT and *n* = 4 KO, *p* < 0.05. **b** Diagram of how mass spectrometry data (mass spec) were used to compile lists of potential biomarker candidates **c**) Schema of PF treatment (top) western blots of Hk1 and Ras normalized to Transferrin receptor (Tffr) (middle) and quantification (bottom) (Hk1 KO Veh *n* = 8, Hk1 KO PF *n* = 5, *p* = 0.81; Ras KO Veh *n* = 8, Ras KO PF *n* = 10, *p* = 0.008). **d** Schema of metformin treatment (top) western blots of Hk1 and Ras normalized to Transferrin receptor (Tffr) (middle) and quantification (bottom). (Hk1 KO Veh *n* = 6, Hk1 KO Met *n* = 8, *p* = 0.24, Ras KO Veh *n* = 7, KO Met *n* = 5, *p* = 0.03) All graphs are represented as mean ± SEM. Statistical analyses using Student’s *t*-test. Outliers were determined a priori as being 2 SD outside of the mean or if the signal to noise ratio was too low on the western blots. Whole blood has high background because of the presence of IgGs and therefore some blots had higher background than brain resulting in the need for higher cohort size to ensure the minimum number of final data points

First we asked whether de novo protein synthesis in whole blood in FXS model mice was affected in a similar manner to brain. Using an azide-alkyne cycloaddition based “click chemistry” protocol, we measured de novo protein synthesis in trunk blood of wild-type mice and *Fmr1* KO littermates and noted a 30% net increase in de novo protein synthesis in the *Fmr1* KO blood, consistent with altered net de novo translation measured in *Fmr1* KO brains (Fig. 4a). Therefore, because protein synthesis is disrupted in blood as well as brain, blood is a logical place to examine proteins affected by FXS for future biomarker efforts.

We hypothesized that the proteins dysregulated in the *Fmr1* KO mouse brain also could be dysregulated in blood and would therefore be potentially useful as biomarkers because (1) FMRP is expressed in blood cells; (2) protein synthesis is disrupted in *Fmr1* KO blood; and (3) some brain proteins are also present in blood. To identify which proteins would be of most interest to measure correlative efficacy, we examined proteins in the most enriched GO clusters under steady state and proteins that changed in *Fmr1* KO mice, in response to DHPG, and were previously measured in blood in any fraction (genecards.org). In addition, we prioritized proteins with reliable antibodies that were not raised in mice (which would lead to non-specific signals; Fig. 4b). We then asked whether any of these markers in peripheral tissue could be useful in monitoring efficacy of a therapy for FXS, as human clinical trial samples are not readily available. We treated *Fmr1* KO mice and their wild-type littermates using three different treatment regimens that affect signaling via different pathways that have been implicated in FXS: mTORC1-S6K1 (the S6K1 inhibitor PF-4708671;“PF”), GSK-3β (lithium; “Li”), and ERK (metformin; “Met”) and analyzed the response of two markers associated with cell membrane and biosynthesis: Ras and HK1, respectively. Due to the difficulty in blood collection and western blotting, outliers were more common than in brain tissue. Outliers were determined a priori as being 2+ standard deviations outside the mean or where the western blotting signal was indistinguishable from the blot background (details provided in Methods).

Adult male wild-type and *Fmr1* KO mice were treated with 25 mg/kg PF-4708671 using a dosing regimen previously shown to normalize behavior, synaptic function, and steady state translation in *Fmr1* KO mice (Fig. 4c; ref. ^23^). HK1 and Ras were reliably detected (Fig. 4c, HK1 KO Veh *n* = 8, KO PF *n* = 5, Ras KO Veh *n* = 8, Ras KO PF *n* = 10, *p* = 0.008), and there was a significant increase in Ras following PF treatment, but not HK1 (Fig. 4c, WT shown in Supplementary Fig. 6). These findings suggest that Ras correlates with PF drug efficacy in *Fmr1* KO mice. We then asked whether changes in the two biomarker candidates correlated with the efficacy of other drugs. Wild-type and *Fmr1* KO mice were treated with 300 mg/kg lithium (ip) for 5 days according to a previously established regimen (ref. ^27^, HK1 KO Veh *n* = 3, KO Li, *n* = 5, *p* = 0.07, Ras KO Veh *n* = 3, KO Li *n* = 6, WT shown in Supplementary Fig. 6). We noted a trend level decrease in HK1 (Supplementary Fig. 6), suggesting that more than one treatment can alter the potential biomarkers identified in the BONLAC screens. We also treated wild-type and *Fmr1* KO mice with 200 mg/kg metformin (ip) for 10 days, a paradigm that was shown to correct behavioral, dendritic spine density, and electrophysiological phenotypes (ref. ^28^, Fig. 4e, HK1 KO Veh, *n* = 6, HK1 KO Met, *n* = 8, Ras KO Veh, *n* = 7, KO Met *n* = 5, *p* = 0.03). We noted significant changes in Ras and three different types of responses in HK1, further suggesting that these markers may be valuable and correlate to efficacy, but are treatment-specific. We also investigated whether mGluR5, a canonical FMRP target, changed in blood with PF and metformin, but there was no change with either treatment (Supplementary Fig. 7). Taken together, these data suggest that some of the markers identified by our BONLAC screens could be useful as treatment-specific correlates of efficacy.

**Fig. 5:**
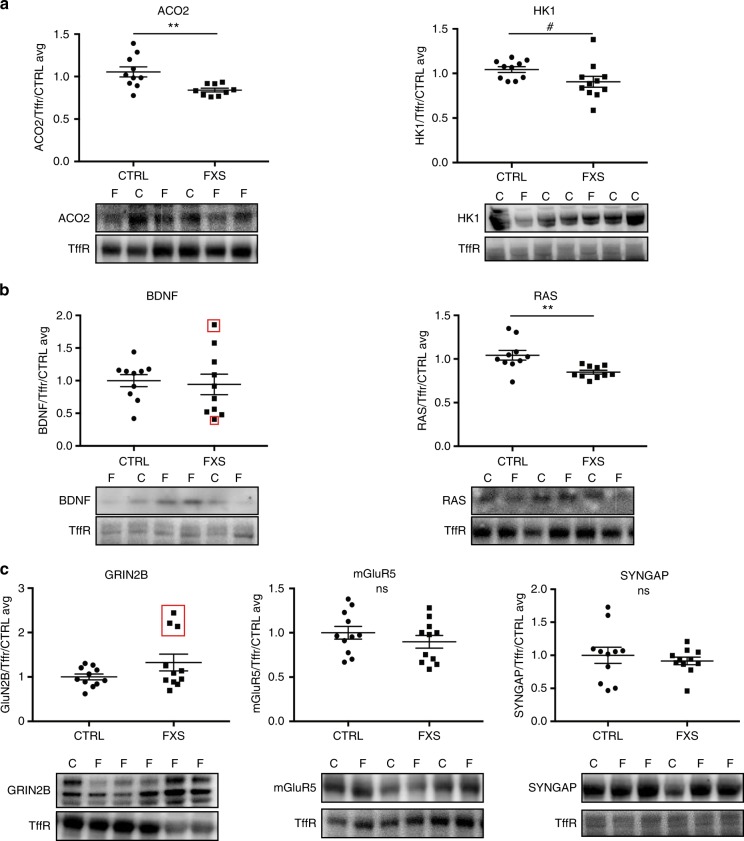
Several BONLAC candidate proteins have altered abundance in human FXS patient plasma. **a** Quantification and western blots of proteins in the biosynthesis pathway in the plasma of healthy human volunteers (CTRL) versus age-matched FXS patients (FXS) * denotes *p* < 0.05, # denotes *p* < 0.10. *n* = 11/group ACO2 *p* = 0.006, HK1 *p* = 0.07. **b** Quantification and western blots of proteins in the cell membrane/intracellular signaling pathway in the plasma of CTRL versus FXS. *n* = 11/group BDNF *p* = 0.35, RAS *p* = 0.007 **c** Quantification and western blots of proteins in the Synapse pathway in the plasma of CTRL versus FXS. *n* = 11/group GRIN2B *p* = 0.13, mGluR5 *p* = 0.32, SYNGAP *p* = 0.76. All blots use transferrin receptor as the loading control for normalization. One dot = one patient or volunteer. Statistical significance was determined using a Student’s *t*-test, the absence of a * or # indicate that the groups are not significantly different. *n* = 11 per group. All graphs are represented as mean ± SEM. Red boxes indicate values that are 2+ standard deviations outside of CTRL average

After identifying two biomarker candidates that changed with treatment in the blood of *Fmr1* KO mice, we asked whether any of the candidates identified by the BONLAC screens were differentially expressed in easily accessible FXS patient tissue. To answer this question, we tested plasma from 11 FXS patients and 11 healthy volunteers from Rush University Medical Center. We expanded the list from HK1 and RAS to seven total proteins identified in the BONLAC screens in the *Fmr1* KO mice and in the top GO categories of biosynthesis proteins, cell membrane proteins, intracellular signaling proteins, and synapse proteins: ACO2, HK1, BDNF, RAS, GRIN2B, mGluR5, and SYNGAP. The previously frozen plasma was thawed, lysed, and analyzed by western blot (Fig. 5a–c). The number of patients was selected based on previous outcome measures in FXS clinical studies^29–31^. We noted significant population differences between healthy volunteers (control) and FXS patients in ACO2 and RAS, with a trend level difference in HK1 with all three proteins having decreased expression compared to controls (Fig. 5a, b). In addition, GRIN2B and BDNF had distinct subpopulations of individual FXS patients with protein expression levels 2+ standard deviations outside the average for controls (3 patients in GRIN2B and 2 in BDNF, red boxes), with additional patients between 1 and 2 standard deviations outside average for controls (2 in GRIN2B and 3 in BDNF) (Fig. 5b, c). mGluR5 and SYNGAP did not demonstrate any population differences, although 6 FXS patients were between 1 and 2 standard deviations outside control average in mGluR5 expression (Fig. 5c). When expression of these proteins is considered separately in each patient, the pattern of expression is at least slightly different (Supplementary Fig. 8), potentially due to differences in genetic, epigenetic, and environmental backgrounds. Together these data underscore (1) the conserved disruption of multiple proteins in FXS human patient blood that were identified in the BONLAC screens in mouse brain and (2) that human patients have different “barcodes” or expression patterns that could inform future subpopulation identification efforts for clinical trials.

## Discussion

The current model of FXS pathology postulates that loss of FMRP-mRNA interactions disrupts synaptic activity-induced translation, leading to aberrant expression of key synaptic proteins and changes in neuronal output and behavior. Inherent in this model is the assumption that the hallmark disruption of protein synthesis is activity-dependent and that steady state differences are similar to those seen with activity. To better understand the relationship between aberrant protein synthesis and FXS pathology, we started with the hypothesis that the *Fmr1* KO de novo proteome would differ from the wild-type proteome, based on previous studies demonstrating elevated protein synthesis in FXS model mice and FXS human tissues. Interestingly, the disruption in protein synthesis in FXS model mice was observed at steady state and in response to mGluR stimulation. Furthermore, one of the main functional clusters of proteins enriched for dysregulation was metabolic function (biosynthesis, Fig. 1). This observation provides strong support to the aberrant metabolomics that have been noted in FXS^32,33^. In particular, the altered regulation of Ras and HK1 in both neural and peripheral tissue represent a strong implication in their role in FXS pathology and new avenues for further investigation.

Notably, we observed that few of the proteins with altered de novo expression at steady state and in response to group I mGluR stimulation were FMRP targets (Fig. 3b), which is consistent with a recent study using TRAP in excitatory neurons in hippocampal area CA1 and departs from the common hypothesis that mainly FMRP targets would exhibit altered translation^34^. There are several potential reasons for this difference, but it is worth noting that FMRP binds mRNA in a variety of functions, not just during protein synthesis. Therefore, not all FMRP-binding partners are necessarily involved the translational process. It also should be noted that several of the small number of FMRP targets we did identify, such as Syngap and mGluR5, are commonly linked to FXS pathology, and therefore our data are consistent with previous studies^35–37^. In addition, although our data provide a glimpse into dysregulated de novo protein synthesis within a 5-h time window in mature hippocampal tissue, the possibility exists that other targets are synthesized earlier in development, in a different temporal window, or in a specific subtype of neurons that was not measured with BONLAC. However, the low number of FMRP-binding target mRNAs that correlated with alterations in newly synthesized proteins in FXS model mice suggests that there are additional under characterized functions of FMRP and regulation of the mRNA targets before they arrive at the ribosome or of their degradation after they are synthesized.

The inability to identify subgroups of FXS patients that will respond to a drug has hindered therapeutic efforts. Approaches used in many basic research studies cannot be translated to meaningful clinical use because of non-transferrable techniques or methodologies^38^. To translate our BONLAC screen in the brains of FXS model mice into clinically-relevant targets for biomarkers, we determined whether the candidate proteins could be identified in blood and asked whether: (1) they are expressed and could be detected reliably, (2) the expression levels of the proteins were sensitive to treatments that have been shown to affect behavior in *Fmr1* KO mice, and (3) differential expression of the proteins could be detected in FXS patients (Figs 4 and 5). We determined that overall de novo protein synthesis is altered in the whole blood of *Fmr1* KO mice (Fig. 4a), laying the foundation for further analyzing changes in specific proteins in blood (Fig. 4c–e). We then demonstrated that treatment of *Fmr1* KO mice with three drugs previously shown to reverse phenotypes rescued the levels of HK1 and/or Ras in whole blood, nominally establishing them as lead biomarker candidates to be considered for efficacy measures in the future. HK1 and Ras responded in a pathway-specific manner to the drugs. The PF-4708671 experiments suggest a reciprocal relationship between Ras and S6K1, which is notable given the regulation of S6K1 by ERK 1/2 (Fig. 4c)^39^. The effect of lithium on HK1 may be linked to the actions of lithium on metabolism via GSK3β and glycogen synthesis. Lithium also decreases Ras activity, but it appears that it does not alter the abundance of Ras^40,41^. Similarly, metformin also has been shown to inhibit Ras and HK1 activity, but previous studies have shown that it does not change the abundance of HK1^42,43^. To address this possibility, we examined a larger panel of the potential biomarkers and found that three, HK1, RAS, and ACO2, exhibit clear population differences in abundance between control and FXS patients, whereas candidates such as Grin2B, BNDF and mGluR5 show stratified expression levels (Supplementary Fig. 8).

Interestingly, BDNF increased with acamprosate treatment in FXS patients, but it did not fully correlate to efficacy, which could potentially be explained by the observation here that BDNF levels are not disrupted in every FXS patient^44^. The proteins we chose to validate in this study were limited as a proof of concept to demonstrate the possibility of bridging basic biology research to the clinic. One should also note that it is unlikely that the same targets would respond to every treatment for FXS and may be different in blood fractions other than in plasma. Thus, more refinement will need to be performed for larger scale biomarker studies. However, even with those caveats, this graded refinement of targets argues for the soundness of the approach of identifying neural proteomic candidates generated in FXS model mice and validating them in blood, which is critical for potential future clinical utility. In contrast to previous studies, we demonstrated that our method of identifying key targets disrupted by the loss of FMRP in the brain of FXS model mice can be detected in and are altered in human FXS plasma and respond in accessible tissue with potential treatment paradigms.

In summary, we have provided direct evidence that de novo proteomic alterations in the hippocampus of FXS model mice in both steady state and activity-dependent translation are altered, but the proteins that are synthesized inappropriately differ between these conditions. We validated several candidate proteins and then examined them in mouse blood in response to drug treatments. We proceeded to determine whether several of the proteins could be biomarkers in FXS and noted differences between FXS patients and healthy controls that occurred in a protein-specific manner. The subtle variations within the individual FXS protein targets may help determine patient-stratification and better approaches to biomarker development to make future clinical trials more effective. Together, these data support a refined hypothesis of multi-tiered protein synthesis disruption across tissues in FXS and a probable method of future biomarker discovery.

## Methods

### Animals

*Fmr1* knockout mice were bred and maintained as described in 23 by crossing female X*Fmr1*+X*Fmr1*− with *XFmr1*+/Y male mice on a C57/Bl6 background (originally sourced from Jackson labs, ME, USA). The colony was routinely backcrossed to wild-type C57/Bl6 mice to prevent inbreeding-related issues. All procedures were in accordance with protocols approved by the New York University Animal Welfare Committee and followed the NIH Guidelines for the care and use of animals in research or the guidelines of the Institute for Stem Cell Biology and Regenerative Medicine. All animals were kept in mixed housing of wild-type and KO animals.

### Slice preparation and slice pharmacology

400 µm transverse slices were prepared from 10 to 12 week old littermate mice as described previously^16,22^. Slices were allowed to recover in ACSF, and then stimulated with (R,S)-3,5-dihydroxyphenylglycine (DHPG; Abcam ab120020) for the times shown in the timeline schematic (Figs 1a, 2b). DHPG was dissolved in ACSF to make a 50 mM master stock. In all cases, slices were flash frozen and then processed for subsequent applications. All experiments were done from littermate and cagemate yoked pairs. The experimenter was blind to genotype. Analyses were performed by a separate experimenter who was blind to all genotypes and treatment conditions.

### Western blotting

All western blotting procedures were carried out as described previously^22^ and analysis was performed blinded by an investigator not involved in the blot generation. The detailed table of the commercial sources of the antibodies and their dilutions is in the Supplementary Methods. Western blots were considered a priori only if the band of interest had a positive value above background and the overall value with background subtracted and normalized was within 2 standard deviations of the group.

### BONCAT and AHA pulldowns

AHA was synthesized as previously described^45^ and purified to >95% as monitored by 1H NMR. For detailed description of the optimization of BONCAT protocols for tissue slices and immunoprecipitations for de novo peptides, see^16^. SILAC amino acids (13C6–15N2-lysine, 13C6-15N4-arginine (Lys8/Arg10) and D4-lysine/13C6-arginine (Lys4/Arg6)) were obtained from Cambridge Isotope Laboratories, MA, USA in concentrations previously described^16^.

For BONCAT, flash frozen sections were thawed and sonicated in lysis buffer^22^ before performing cycloaddition conjugations at room temperature overnight according to the specifications of the manufacturer (Click-iT Protein labeling kit, Invitrogen/Thermo Fisher Scientific, CA, USA; ref. ^16^). For each sample, 30 µg of protein was loaded on 4–12% Bis-Tris gradient PAGE gels (Invitrogen) and followed by transfer onto nitrocellulose membranes (GE Healthcare, UK). Membranes were blocked with either 5% BSA, 0.25% I-block, or 5% non-fat skim milk and probed with 1:2000 anti-biotin (Abcam) overnight at 4 °C. The anti-biotin signal was developed using 1:5000 anti-goat HRP (Jackson Immunoresearch, PA, USA) or anti-biotin (Sigma Aldrich, MO, USA). Blots were developed using either standard or enhanced chemiluminescence detection (GE Healthcare, CT, USA) and imaged using ProteinSimple FluorChemE (ProteinSimple, CA, USA) imaging system. Exposures were set to obtain signals in the linear range and then normalized by total protein and quantified via densitometry by a blinded investigator.

For AHA pulldowns, protein lysates tagged with AHA underwent cycloaddition reactions as described (Fig. 1a,  e). The lysates were diluted with PBS to a final volume of 1 ml and excess biotin was removed by dialysis against PBS overnight at 4 °C. AHA-biotin-tagged proteins were precipitated using Streptavidin Agarose resin (Thermo Scientific, IL, USA) overnight at room temperature. Total protein input of 600 μg was used for cytosolic or membrane-bound proteins in the pulldowns. Washes and elution were performed as described previously^16^. A mixture of Laemmli buffer and beads was added to the SDS-PAGE gel and lysates were analyzed by western blot.

### BONLAC, C-score and gene ontology analyses

Combinatorial BONCAT and SILAC mass spec screen (BONLAC) was carried out as described previously with 3 runs per condition and hippocampal slices pooled from 3 animals making up each group per run (*n* = 3 biological replicates made up of 3 pooled animals/ treatment condition for each run each treatment has 3 biological replicates—8–9 animals/treatment in total; ref. ^16^). SILAC conditions (heavy or medium) were alternated between runs to ensure there was no labeling bias. BONCAT enrichment procedures including cell lysis and tryptic digestion were performed using a kit (Click-iT Protein Enrichment Kit, Invitrogen) with minor modifications. Resulting newly synthesized proteins were analyzed by nanoflow LC-MS/MS (Q-Exactive), and data analyzed using MaxQuant. See Supplementary Methods for full details. All raw mass spectrometry data are deposited at ftp://MSV000081061@massive.ucsd.edu.

MaxQuant normalized H/M ratios (heavy vs. medium isotopes) were used for quantitative analysis. Ratios were inversed for experiments with reversed isotopic labeling. Total intensities were calculated as the sum of the heavy and medium ion intensities, as reported by MaxQuant. For C-score, the statistical significance of the log-2 transformed ratios and total intensities was analyzed by the Perseus software (v. 1.1.1.34) using the Significance_B option and the Benjamini–Hochberg correction to account for multiple hypothesis testing. Proteins with *p* ≤ 0.3 were selected and then scored for consistency. Consistency criteria was only those proteins measured at least twice and those that were consistently up or downregulated each time they were detected. Only those proteins that met C-score and/or consistency criteria were deemed de novo protein candidates. Gene ontology (GO) analysis was performed using the Database for Annotation, Visualization and Integrated Discovery (DAVID 6.8)^46^. Candidate proteins were added as the gene list, and the background was considered as all proteins measured in hippocampal brain slices. Clusters with FDR values of less than 0.05 were considered, with the most focus given to candidates that appeared multiple times in clusters. Visualization of the gene ontology for steady state was provided by the BinGO plug-in for cytoscape using the candidate list as a gene list (Maere et. al. 2005). Groups with *p* < 0.05 were included.

### Mouse blood protein synthesis assay

Adult male *Fmr1* KO and wild-type littermates were euthanized by cervical dislocation, decapitated, and their trunk blood was collected in heparinized tubes and labeled with AHA for 1 h and snap frozen on dry ice. Samples then were lysed, processed, and underwent click chemistry to add biotin to newly synthesized proteins. Samples then were analyzed by western blot using streptavidin-HRP and transferrin receptor as a loading control, as it is ubiquitous in blood, and often used as a loading control for cell-based assays.

### Drug administration

All i.p. injections were performed at the same time of day by the same experimenter who was blind to genotypes. PF-4708671 was obtained from Sigma, MO, USA. Littermate matched mice underwent 10 days of i.p injections of 25 mg/kg PF-4708671 in a vehicle consisting of saline  +  5% v/v of Tween-80 (Sigma, USA) as previously described^16^. Lithium citrate (Sigma, USA) in saline or saline was injected at 300 mg/kg ip for 5 days per a previously established paradigm^27^. Metformin (Tocris, USA) was prepared in saline and injected at 200 mg/kg and administered for 10 days i.p. at 200 mg/kg while saline vehicle only was administered to littermates^28^. On the last day of treatment, mice were killed by cervical dislocation and the hippocampus was quickly dissected to yield whole hippocampal lysates. Simultaneously, trunk blood also was collected to monitor peripheral levels of proteins. Changes in phosphorylation in hippocampal lysates from treated animals were used to verify drug efficacy for PF (S6), Lithium (GSK-3B), metformin (ERK). All drug treatments were administered on 2–3 cohorts of mice, some of which were months apart. To combat antibody variability over time, all blots were run with a WT vehicle from that cohort and then protein of interest signal was normalized to the average WT vehicle signal for each blot before being combined and analyzed together.

### Human FXS patient studies

All subjects or guardians provided voluntary informed consent as appropriate in accordance with Rush University Medical Center IRB regulations. Participants were Caucasian with a DNA-confirmed FMR1 full mutation of over 200 CGG repeats (FXS) or healthy volunteers (CTRL) with normal FMR1 alleles. FXS patients were aged 16–35 with typical FXS phenotypes, an IQ range of 40–50 and adaptive behavior standard score range of 31–56. CTRL subjects were aged 22–59 with a normal IQ and were free of neuropsychiatric conditions. Plasma was collected and isolated using standard methods and snap frozen immediately. It was later shipped on dry ice to New York University where it was thawed and immediately lysed with Laemmli buffer at room temperature then analyzed via western blot. Western blot images were obtained using a Protein Simple FluorChem E with chemiluminescence and quantified blindly by a separate investigator using Image J. All proteins were normalized to transferrin receptor and the average signal of the control subjects. A subset of western blots was blindly repeated at least once to ensure there was no sample-to-sample variation. Most antibodies were also spot checked for specificity by running brain and blood tissue on the same Western to ensure protein size and signal was similar. There were no correlations with protein abundance and date of collection, age, or sex. Samples we analyzed by a student t-test assuming unequal variance, a priori criteria for exclusion was 2+ standard deviations outside the mean of the group.

### Statistical analysis

All statistical analysis was performed as described in each experiment. Unless otherwise noted, statistics were performed using Microsoft Excel, SPSS or GraphPad Prism. A priori outliers were determined as those with values 2+ standard deviations outside the mean of the group or if a technical error occurred during the experiment.

## Supplementary information

Supplementary Information

Description of Additional Supplementary Files

Supplementary Data 1

Supplementary Data 2

Supplementary Data 3

Supplementary Data 4

Supplementary Data 5

Source Data

## Data Availability

All raw proteomic data can be found at https://massive.ucsd.edu/ProteoSAFe/datasets.jsp#%7B%22query%22%3A%7B%7D%2C%22table_sort_history%22%3A%22createdMillis_dsc%22%7D (MassIVE ID MSV000081061). DAVID original data can be found at https://drive.google.com/open?id=169k493D4SJKSLjOst-oSmR19rScT5o8w. The source data underlying Figs. 1e, 2a, 4a, 4c-d, 5a-c are provided as a Source Data file. All other data is available from the corresponding authors upon request.
