# Supplementary material for: Altered steady state and activity-dependent de novo protein expression in fragile X syndrome
Source: Nat Commun. 2019 Apr 12;10:1710. doi: 10.1038/s41467-019-09553-8 (PMC6461708; doi:10.1038/s41467-019-09553-8)
Supplement: Supplementary file 1 — Supplementary Information [file 41467_2019_9553_MOESM1_ESM.pdf]

Supplementary Information
(Supplementary Figures and Methods) For:

Altered steady state and activity-dependent *de novo* protein expression in fragile X syndrome

Bowling, Bhattacharya et al.

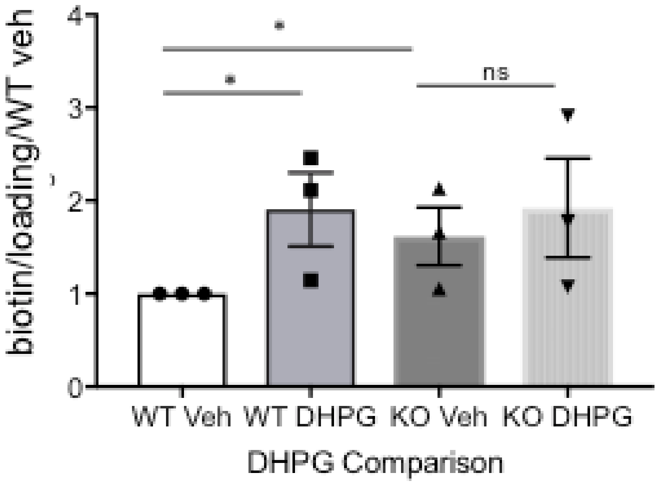

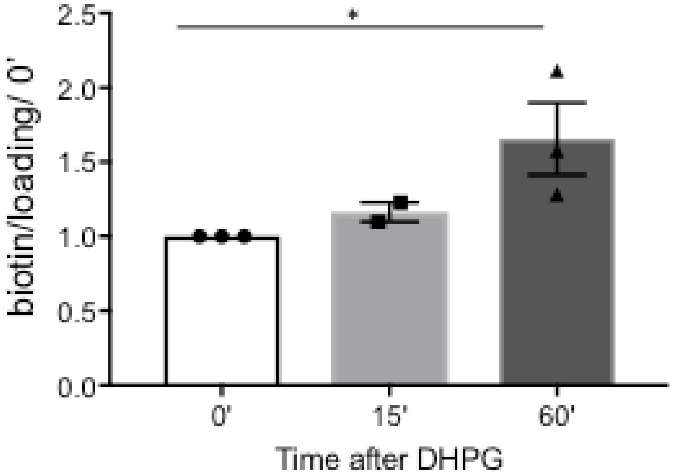

Supplementary Figure 1. DHPG treatment results in increased *de novo* protein synthesis in wild-type, but not *Fmr1* knockout mice. Acute adult hippocampal slices were prepared and treated for 10 minutes with 100 μM DHPG followed by a 1-hour recovery (top panel,n=3 mice/group, WT veh vs WT DHPG p = 0.04, WT veh vs KO veh p = 0.06, KO ve vs KO D p = 0.32). Wild-type slices were treated with DHPG as described in top panel and were allowed to recover for 0', 15', or 60' recovery (bottom panel). Slices were then lysed, cycloadded and probed for changes in biotin abundance via western blots (0' and 60' n=3 mice/group, 3 slices per mouse p =0.05; 15' n=2 mice/group). Top panel demonstrates that there is a DHPG-induced increase in protein synthesis in wild-type (WT) but not Fmr1 knockout (KO) mice. All graphs are represented as mean ± SEM. All significance determined using a Student's t-test.

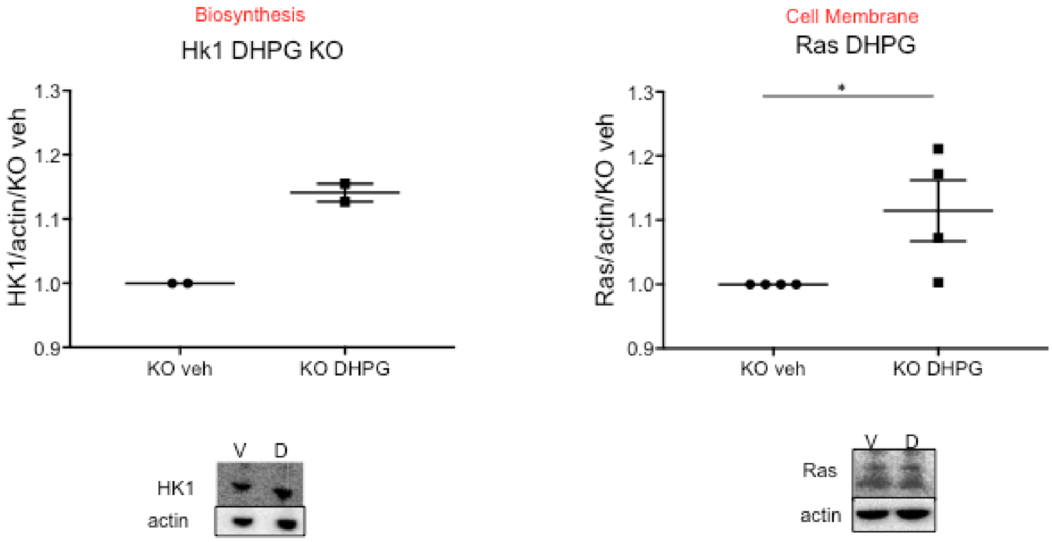

Supplementary Figure 2. DHPG treatment increases Biosynthesis (Hk1) and Cell membrane (Ras) proteins in *Fmr1* knockout mice as predicted by the BONLAC screen. Acute adult hippocampal slices were prepared and treated for 10 minutes with100 μM DHPG followed by 1-hour recovery. Slices were then lysed and probed for changes via western blot (n=2 animals for HK1, n=3 animals for RAS). All graphs are represented as mean ± SEM. * indicates p=0.05 using a Student's t-test. Statistics were not performed on HK1 as n=2, but both experiments showed the same trend that supported the proteomic data.

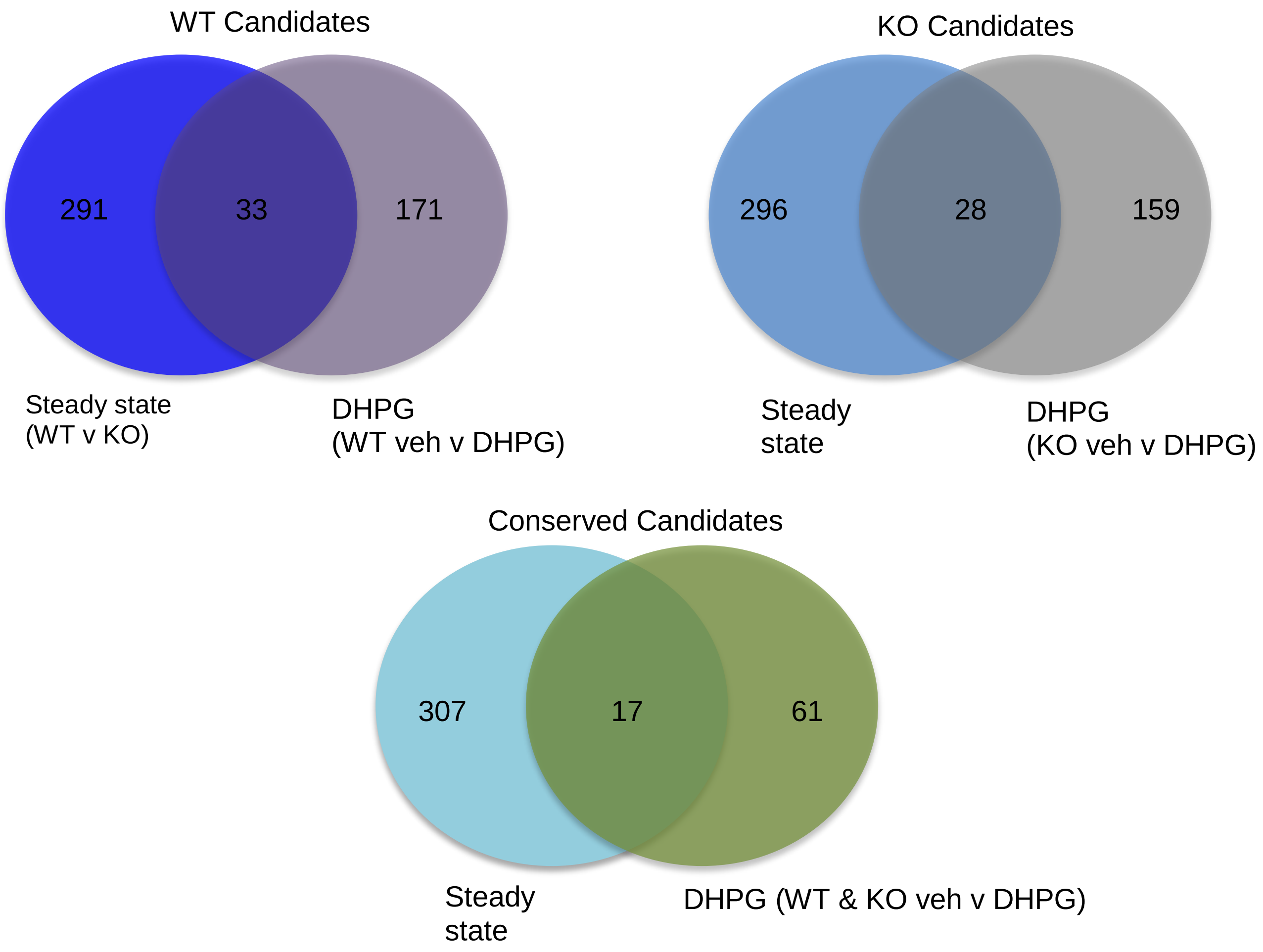

Supplementary Figure 3. There is minimal overlap between candidates identified in the steady state versus DHPG-stimulated *de novo* protein synthesis experiments. We compared the total number of candidates in steady state and DHPG-stimulated conditions for wild-type (WT), *Fmr1* knockout (KO) and conserved between WT and KO (C) mice. In each case there was overlap in candidates, but it minimal compared to the total number of candidates represented.

Comparing the overlap of Ensembl gene IDs of all proteins enriched (up and down) in KO steady state experiment with the Ensembl gene IDs of all proteins measured in Gomez-Serrano et al. (2016) – using 53946 genes available in Ensembl as the background

Detailed information about this GeneOverlap object:
listA size=319, e.g. ENSMUSG00000003072 ENSMUSG00000004032 ENSMUSG00000036499
listB size=2125, e.g. ENSMUSG00000026817 ENSMUSG00000036932 ENSMUSG00000023262
Intersection size=169, e.g. ENSMUSG00000003072 ENSMUSG00000036499 ENSMUSG00000039682
Union size=2275, e.g. ENSMUSG00000003072 ENSMUSG00000004032 ENSMUSG00000036499
Genome size=53946
Contingency Table:

	notA	inA
notB	51671	150
inB	1956	169

Overlapping p-value=6.1e-149
Odds ratio=29.8
Overlap tested using Fisher's exact test (alternative=greater)
Jaccard Index=0.1

Conclusion: the protein candidates from KO Steady state are largely represented in the total proteins measured in Gomez-Serrano et al. 2016.

Comparing Ensembl gene IDs of all proteins enriched (up and down) in KO steady state experiment with the Ensembl gene identifiers of a subset of differentially abundant proteins in the "Age" test in Gomez-Serrano (2016) – using a total set of 5000 proteins as background

listA size=319, e.g. ENSMUSG00000003072 ENSMUSG00000004032 ENSMUSG00000036499
listB size=88, e.g. ENSMUSG00000006574 ENSMUSG00000019843 ENSMUSG00000015854
Intersection size=8, e.g. ENSMUSG00000000568 ENSMUSG00000052397 ENSMUSG00000049539
Union size=399, e.g. ENSMUSG00000003072 ENSMUSG00000004032 ENSMUSG00000036499
Genome size=5000
Contingency Table:

	notA	inA
notB	4601	311
inB	80	8

Overlapping p-value=0.2
Odds ratio=1.5

Overlap tested using Fisher's exact test (alternative=greater)
Jaccard Index=0.0

Conclusion: the protein candidates from KO Steady state are not represented in the enriched/candidate proteins measured in Gomez-Serrano et al. 2016

Comparing the overlap of Ensembl gene IDs of all proteins enriched in the DHPG treatment BONLAC experiment with the Ensembl gene IDs of all proteins measured in Gomez-Serrano (2016)

Detailed information about this GeneOverlap object:
listA size=470, e.g. ENSMUSG00000021087 ENSMUSG00000034187 ENSMUSG00000034088
listB size=2125, e.g. ENSMUSG00000026817 ENSMUSG00000036932 ENSMUSG00000023262
Intersection size=264, e.g. ENSMUSG00000021087 ENSMUSG00000034187 ENSMUSG00000007815
Union size=2331, e.g. ENSMUSG00000021087 ENSMUSG00000034187 ENSMUSG00000034088
Genome size=53946
Contingency Table:

	notA	inA
notB	51615	206
inB	1861	264

Overlapping p-value=2.5e-243
Odds ratio=35.6
Overlap tested using Fisher's exact test (alternative=greater)
Jaccard Index=0.1

Conclusion: the protein candidates from the DHPG BONLAC experiments are largely represented in the total proteins measured in Gomez-Serrano et al. 2016*.*

Comparing the overlap of Ensembl gene IDs of all protein candidates in the DHPG treatment BONLAC experiment with the Ensembl gene identifiers of a subset of differentially abundant proteins in the "Age" test in Gomez-Serrano (2016)

Detailed information about this GeneOverlap object:
listA size=470, e.g. ENSMUSG00000021087 ENSMUSG00000034187 ENSMUSG00000034088
listB size=88, e.g. ENSMUSG00000006574 ENSMUSG00000019843 ENSMUSG00000015854
Intersection size=3, e.g. ENSMUSG00000024608 ENSMUSG00000026895 ENSMUSG00000037710
Union size=555, e.g. ENSMUSG00000021087 ENSMUSG00000034187 ENSMUSG00000034088
Genome size=5000
Contingency Table:

	notA	inA
notB	4445	467
inB	85	3

Overlapping p-value=0.99
Odds ratio=0.3
Overlap tested using Fisher's exact test (alternative=greater)
Jaccard Index=0.0

Conclusion: the protein candidates from the DHPG BONLAC experiments are not represented in the enriched/candidate proteins measured in Gomez-Serrano et al. 2016

Summary: The bulk of our candidates from the BONLAC screens are present in other proteomic studies, but not enriched suggesting specificity of the proteins identified to the specific conditions in question (FXS vs. DHPG response vs. metabolic disruption/obesity).

Supplementary Figure 4. Comparison of BONLAC candidates to an unrelated proteomic dataset. Candidates from the BONLAC screens (Figures 1 and 2) were compared with the total proteins measured and the enriched candidates from a “random” proteomics paper on obesity (Gomez-Serrano et al, 2016) to determine whether the overlap observe in the ASD and FMRP databases were specific. Although the same proteins were measured in both the BONLAC and the random dataset, the same proteins were not enriched or identified as candidates, therefore further suggesting that protein candidates were specific to the measurement (FXS and DHPG), and that the overlap with other databases involving FXS and ASD was not coincidental.

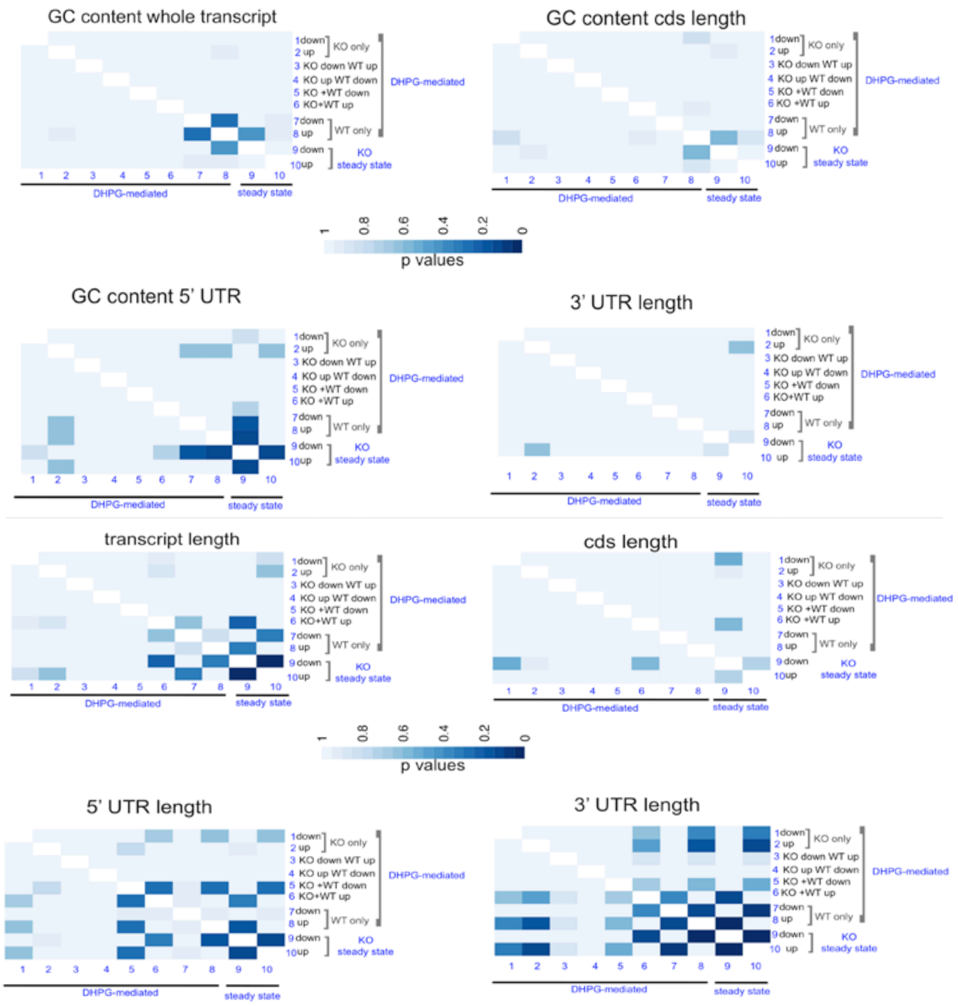

		steady state	KO dhpg	WT dhpg
predicted length	Transcript	0.0012	0.5416	0.0828
	CDS	0.0555	0.3427	0.7977
	5'UTR	0.0076	0.0263	0.3057
	3'UTR	0.0023	0.8028	0.0455
predicted GC content	Transcript	0.3731	0.8691	0.0312
	CDS	0.4956	0.1839	0.3756
	5'UTR	0.0104	0.8787	0.2792
	3'UTR	0.8026	0.7421	0.0316

Supplementary Figure 5. Kruskal-wallis significance values obtained by comparing mRNA properties of upregulated and downregulated candidates from BONLAC screens. Vertical axis lists the mRNA property like length or GC content. Transcript= total mRNA length including introns, CDS= coding sequence, 5'UTR= upstream 5' untranslated region, 3'UTR= downstream 3' untranslated region. Entries in blue display p value (gradiation noted in the middle of the plots). Frequency distribution plots of mRNAs in up and down regulated candidates for GC content (color denotes P value). Frequency distribution plots of mRNAs in regulated candidates for each mRNA property that showed significant differences. These include transcript length, 5' and 3' UTR for basal wild-type (WT) versus Fmr1 knockout (KO) BONLAC candidates and only 5' and 3' UTR lengths for DHPG-stimulated candidates.

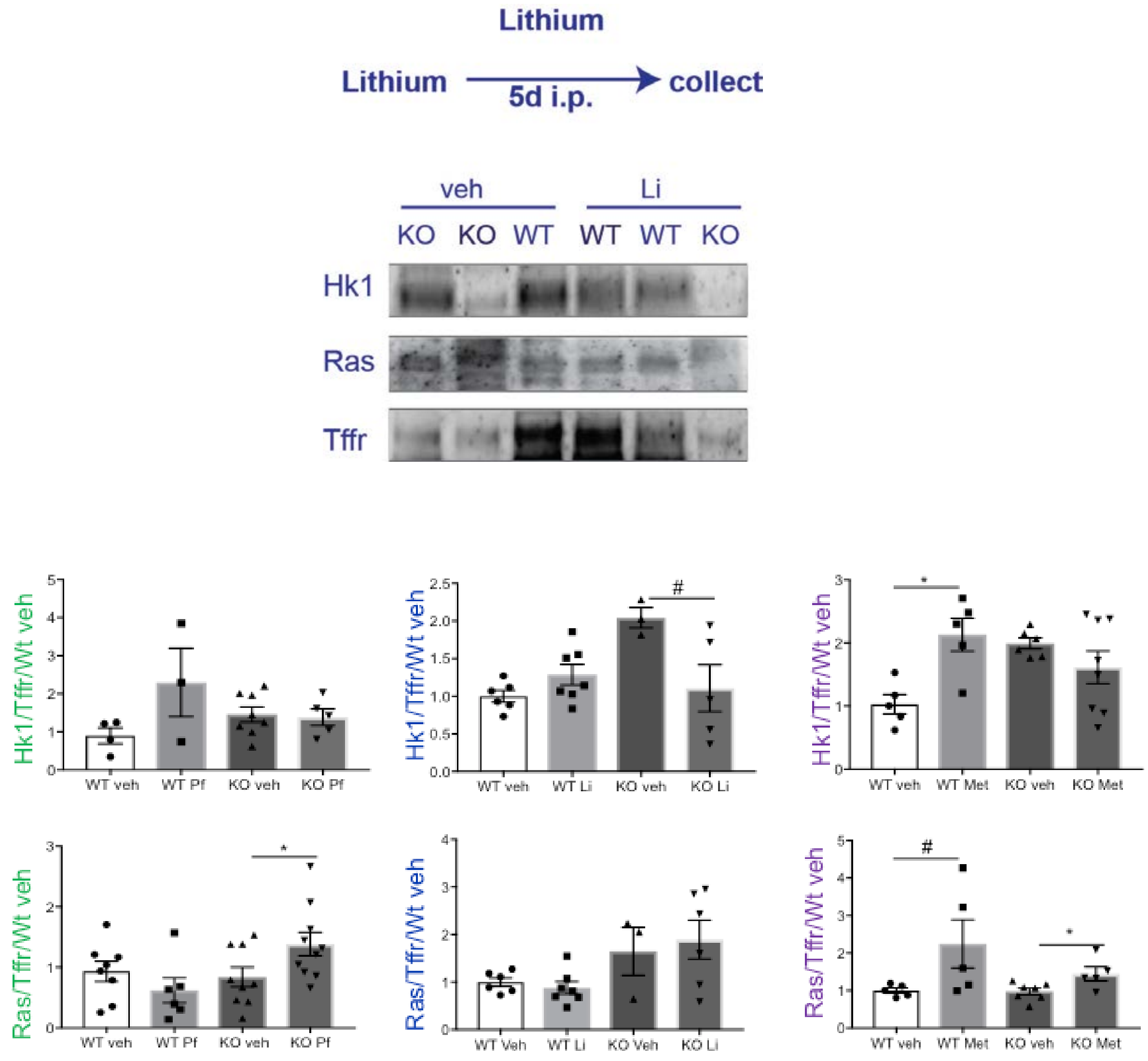

Supplementary Figure 6. Effects of ameliorative treatments in the whole blood of *Fmr1* knockout mice and their wild-type littermates. Adult male mice were treated three ameliorative treatments: PF (left), Li (Top and center), and Met (right) and probed for 2 markers – Hk1 and Ras. Each graph depicts wild-type (WT) versus *Fmr1* knockout (KO) (Figure 4 contains only the KO portion of the graph for S6K1i and Metformin). All data is mean ± SEM. Data was analyzed using a Student's t-test for differences between KO veh and drug treatment and WT vehicle vs WT drug treatment HK1 Pf WT veh, n=4, WT Pf n=3, KO veh n=8, KO Pf n = 5, Ras WT veh n=8, WT Pf n=6, KO veh n=9, KO Pf n=10, HK1 Li WT veh n =6, WT Li n=7, KO veh n=3, KO Li n=5, Ras Li WT veh n=6, WT Li n=7, KO Veh n=3, KO Li n=6, HK1 Met WT Veh n=5, WT Met n=5, KO Veh n=6, KO Met n=8, RAS Met WT veh n=5, WT Met n=5, KO veh n=7, KO Met=5. PF: RAS KO veh vs KO Pf p = 0.008, Li: HKI KO Veh vs KO Li p=0.07, Met: Ras KO veh vs. KO Met p = 0.03, Hk1 WT veh vs WT Met p = 0.006, Ras WT veh vs WT Met p = 0.09

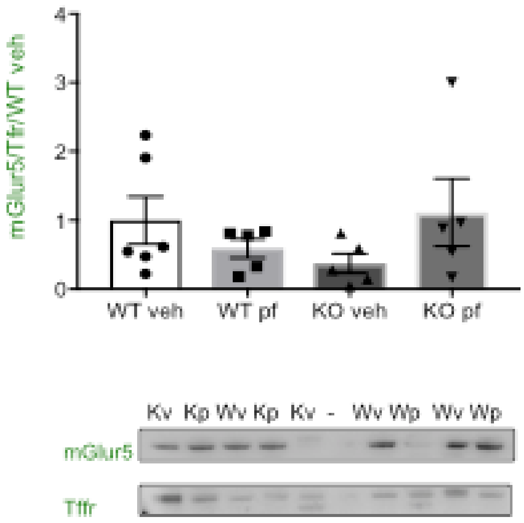

Supplementary Figure 7. Effects of S6K1 inhibitor in the whole blood of *Fmr1* knockout mice and their wild-type littermates for mGluR5. Adult male mice were treated with the S6K1 inhibitor PF-4708671 and analyzed for mGluR5 by western blotting. All data is mean ± SEM. There were no statistical differences between groups. n = 5/group.

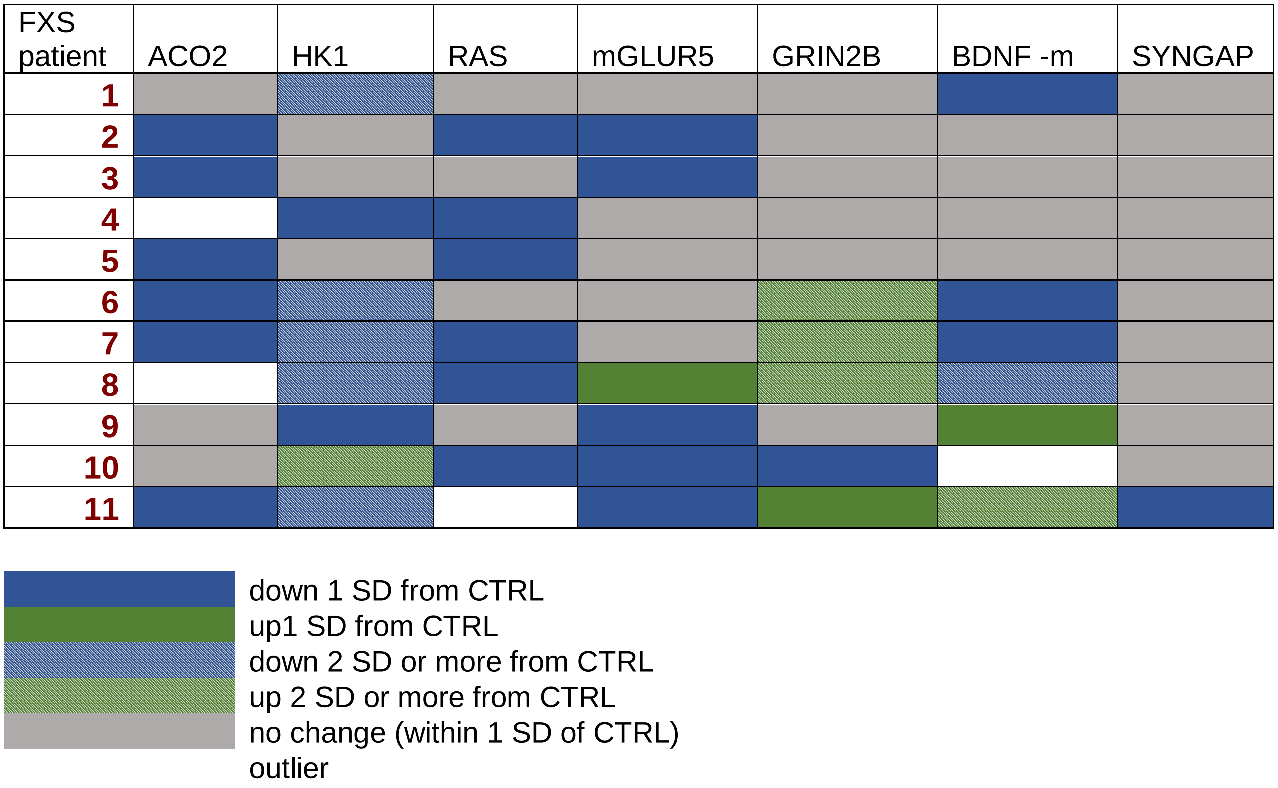

Supplementary Figure 8. Potential "Patient Barcode" examples based on data collected from human patient samples. All data were generated by western blot, normalized to transferrin receptor, then to the average CTRL score. Colors indicate proteins in FXS patients in relation to CTRL average (within 1 standard deviation, more than 1 standard deviation or 2 or more) to demonstrate how much the FXS patient individual protein levels deviate from the control population.

Supplementary Methods

Sample Preparation for Mass Spectrometry

Briefly, after labeling, slices were lysed in buffer containing 8 M urea, 200 mM Tris pH 8, 4% CHAPS, 1 M NaCl, and protease inhibitor cocktail (Sigma). The lysate was sonicated to reduce viscosity. AHA-labeled nascent proteins were covalently coupled to alkyne agarose beads using reagents provided in the kit (Click-iT Protein Enrichment Kit, Invitrogen). Beads were transferred to a SDS wash buffer (1% SDS, 100 mM Tris pH 8, 250 mMNaCl and 5 mM EDTA) and proteins on beads then were reduced with DTT at 70 °C and alkylated with iodoacetamide at room temperature. The beads then were washed sequentially with 100 column volume of the following three buffers to remove non-specific binding proteins: (1) the SDS wash buffer, (2) 8 M urea, and (3) 20% acetonitrile. Immobilized proteins were digested with trypsin on-resin at 37 °C overnight in 25 mM ammonium bicarbonate and the resulting tryptic peptides were desalted using StageTips and dried under vacuum in a SpeedVac device.

Liquid Chromatography and Mass Spectrometry

For LC-MS, a Thermo Scientific EASY-nLC 1000 coupled to a Q Exactive mass spectrometer (Thermo Fisher Scientific) was used. A self packed 75-µm × 25-cm reverse phase column (Reprosil C18, 3 µm, Dr. Maisch GmbH, Germany) was used for peptide separation. Peptides were eluted by a gradient of 3–30% acetonitrile in 0.1% formic acid over 120 min at a flow rate of 250 nL/min at 45°C. The Q Exactive was operated in data-dependent mode with survey scans acquired at a resolution of 50,000 at m/z 400 (transient time = 256 ms). Up to the top 10 most abundant precursors from the survey scan were selected with an isolation window of 1.6 Thomsons and fragmented by higher-energy collisional dissociation with normalized collision energies of 27. The maximum ion injection times for the survey scan and the MS/MS scans were 60 ms, respectively, and the ion target value for both scan modes were set to 1,000,000.

Protein Identification and Quantitation

The raw files were processed using the MaxQuant computational proteomics platform version 1.2.7.0 for peptide identification and quantitation. The fragmentation spectra were searched against the Uniprot mouse protein database (downloaded on Feb 08, 2013), allowing up to two missed tryptic cleavages. Carbamidomethylation of cysteine was set as a fixed modification, and oxidation of methionine and protein N-terminal acetylation, D4-lysine, 13C6-arginine, 13C6-15N2-lysine and 13C6-15N4-arginine were used as variable modifications for the database search. The precursor and fragment mass tolerances were set to 7 and 20 ppm, respectively. Both peptide and protein identifications were filtered at 1% false discovery rate (FDR).

RNA data analyses

RNA properties survey: Consistently changed proteins in wild-type + vehicle or DHPG and KO wild-type + DHPG DHPG/Vehicle treated WT or fmr1 hippocampal slices were further categorized into intersectional lists: WT unique up, WT unique down, WT+KO up, WT+KO down, KO unique up, KO unique down, WT and KO conserved identity but opposite regulation. Features of the canonical transcript of each protein in all lists were obtained from Ensembl using Biomart. These features included whole transcript, coding sequence, 5' untranslated region and 3' untranslated region, lengths (natural log transformed) and GC contents. One-way Anova and Tukey's HSD post-hoc test were used to test for significant differences in any of the features across the different categories. Intra-category (up vs. down) differences were tested using Krukal-Wallis rank sum tests.

Autism Candidate Database

All subsequent steps were performed by an experimenter who was blinded to the contents of the BONLAC gene lists. First, a database was created of known ASD risk genes in previous studies that used either whole-exome or RNA sequencing methods on either ASD patient samples or established ASD models. The database also included previously published FMRP target mRNAs (See Table S3 for citation of all data sources). Next, a literature search on PubMed was performed using the following keyword search to identify relevant articles through their abstracts: ("autism"[Abstract] OR "fragile x syndrome"[Abstract]) AND ("whole exome sequencing"[Abstract] OR

“exome sequencing”[Abstract] OR “RNA sequencing”[Abstract]) From this initial list of 114 hits, 21 final sources were selected using the following criteria: (1) source published within last 5 years; (2) article provided a list of 5 or more genes deemed “candidate genes,” “risk genes,” or something similar; (3) source analyzed ASD patient data or known ASD models with accompanying statistical analyses; and (4) no repetition of first authors. In addition to these sources, we included the SFARI gene list, a commonly referenced and frequently updated assemblage of top ASD risk genes (gene.sfari.org). Once we created our database, a script was written to compare the database to the BONLAC gene lists and keep track of overlaps, which were defined as “full” if the gene name was present in both lists or “partial” if the gene names differed by a final letter/number, as this indicated likely isoforms (e.g. GRIN2A/GRIN2B or TPPP/TPPP3). Overlapping genes underwent gene ontology analysis using the Database for Annotation, Visualization, and Integrated Discovery (DAVID; see Bowling et al., 2016).

Western Blotting- Antibody sources and working concentrations

	**Company**	**Dilution**	**Blocking**
mGluR5	Millipore, AB5676	1:2000	5% BSA in TBST
NMDAR2B/Grin2B	BD Biosciences 610417	1:2000	5% BSA in TBST
SynGap	Cell Signaling Technology D20C7	1:2000	5% BSA in TBST
ACO2	Abcam ab110321	1:1000	5% BSA in TBST after 5% Milk in TBST block
Hk1	Cell signaling 2024	1:1000	5% BSA in TBST after 5% Milk in TBST block
BDNF	Isagen 327-100	1:1000	5% BSA in TBST after 5% Milk in TBST block
HRas/Ras	Cell Signaling Technology 3965	1:1000	5% BSA in TBST
FMRP	Millipore	1:1000	5% milk in

	MAB2160		TBST
Actin	Sigma	1:5000	5% BSA in TBST
Transferrin	Abcam ab84036	1:1000 - 1:2000	5% BSA in TBST after 5% Milk in TBST block
Biotin	Abcam - ab19218	1:2000	5% BSA in TBST
Anti-rabbit HRP	Promega	1:5000	5% BSA in TBST
Anti-mouse HRP	Promega	1:5000	5% BSA in TBST
Anti-goat HRP	Jackson Immunoresearch	1:5000	5% BSA in TBST

References related to Supplemental Methods (In Supplementary Table 5)

1. Ascano, M. Jr, Mukherjee, N., Bandaru, P., Miller, J.B., Nusbaum, J.D., Corcoran, D.L., Langlois, C., Munschauer, M., Dewell, S., Hafner, M., Williams, Z., Ohler, U., & Tuschl, T. FMRP targets distinct mRNA sequence elements to regulate protein expression. *Nature* **492**, 382-386 (2012).

2. Ben-David, E., & Shifman, S. Combined analysis of exome sequencing points toward a major role for transcription regulation during brain development in autism. *Mol Psychiatry* **18,** 1054-1056 (2013).

3. Chang, J., Gilman, S.R., Chiang, A.H., Sanders, S.J., & Vitkup, D. Genotype to phenotype relationships in autism spectrum disorders. *Nat Neurosci* **18**, 191-198 (2015).

4. Codina-Solà, M., Rodríguez-Santiago, B., Homs, A., Santoyo, J., Rigau, M., Aznar-Laín, G., Del Campo, M., Gener, B., Gabau, E., Botella, M.P., Gutiérrez-Arumí, A, Antiñolo, G., Pérez-Jurado, L.A., & Cuscó, I. Integrated analysis of whole-exome sequencing and transcriptome profiling in males with autism spectrum disorders. *Mol Autism* **6**, 21 (2015).

5. Cukier, H.N., Dueker, N.D., Slifer, S.H., Lee, J.M., Whitehead, P.L., Lalanne, E., Leyva, N., Konidari, I., Gentry, R.C., Hulme, W.F., Booven, D.V., Mayo, V., Hofmann, N.K., Schmidt, M.A., Martin, E.R., Haines, J.L, Cuccaro, M.L., Gilbert, J.R., & Pericak-Vance, M.A. Exome sequencing of extended families with autism reveals genes shared across neurodevelopmental and neuropsychiatric disorders. *Mol Autism* **5**, 1 (2014).

6. Darnell, J.C., Van Driesche, S.J., Zhang, C., Hung, K.Y., Mele, A., Fraser, C.E., Stone, E.F., Chen, C., Fak, J.J., Chi, S.W., Licatalosi, D.D., Richter, J.D., & Darnell, R.B. FMRP stalls ribosomal translocation on mRNAs linked to synaptic function and autism. *Cell* **146**, 247-261 (2011).

7. De Rubeis, S., He, X., Goldberg, A.P., Poultney, C.S., Samocha, K., Cicek, A.E., Kou, Y., Liu, L., Fromer, M., Walker, S., Singh, T., Klei, L., Kosmicki, J., Shih-Chen, F., Aleksic, B., Biscaldi, M., Bolton, P.F., Brownfeld, J.M., Cai, J., Campbell, N.G., Carracedo, A., Chahrour, M.H., Chiocchetti, A.G., Coon, H., Crawford, E.L., Curran, S.R., Dawson G, Duketis E, Fernandez BA, Gallagher L, Geller E, Guter SJ, Hill RS, Ionita-Laza, J., Jimenz Gonzalez, P., Kilpinen, H., Klauck, S.M., Kolevzon, A., Lee, I., Lei, I., Lei, J., Lehtimäki, T., Lin, C.F., Ma'ayan, A., Marshall, C.R., McInnes, A.L., Neale, B., Owen, M.J., Ozaki, N., Parellada, M., Parr, J.R., Purcell, S., Puura, K., Rajagopalan, D., Rehnström, K., Reichenberg, A., Sabo, A., Sachse, M., Sanders, S.J., Schafer, C., Schulte-Rüther M, Skuse D, Stevens C, Szatmari P, Tammimies K, Valladares O, Voran A, Li-San, W., Weiss, L.A., Willsey, A.J., Yu, T.W., Yuen, R.K.; DDD Study; Homozygosity Mapping Collaborative for Autism; UK10K Consortium, Cook, E.H., Freitag, C.M., Gill, M., Hultman, C.M., Lehner, T., Palotie, A., Schellenberg, G.D., Sklar, P., State. M.W., Sutcliffe, J.S., Walsh, C.A., Scherer, S.W., Zwick, M.E., Barett, J.C., Cutler, D.J., Roeder, K., Devlin, B., Daly, M.J., & Buxbaum, J.D. Synaptic, transcriptional and chromatin genes disrupted in autism. *Nature* **515**, 209-215 (2014).

8. Egawa, J., Watanabe, Y., Wang, C, Inoue, E., Sugimoto, A., Sugiyama, T., Igeta, H., Nunokawa, A., Shibuya, M., Kushima, I., Orime, N., Hayashi, T., Okada, T., Uno, Y., Ozaki, N., & Someya, T. Novel rare missense variations and risk of autism spectrum disorder: whole-exome sequencing in two families with affected siblings and a two-stage follow-up study in a Japanese population. *PLoS One* **10**, e0119413 (2015).

9. Hoeffer, C.A., Sanchez, E., Hagerman, R.J., Mu,Y., Nguyen, D.V., Wong, H., Whelan, A.M., Zukin, R.S., Klann, E., & Tassone, F. Altered mTOR signaling and enhanced CYFIP2 expression levels in subjects with fragile X syndrome. *Genes Brain Behav* **11**, 332-341 (2012).

10. Hu, V.W., Sarachana, T., Sherrard, R.M., & Kocher, K.M. Investigation of sex differences in the expression of RORA and its transcriptional targets in the brain as a potential contributor to the sex bias in autism. *Mol Autism* **6**, 7 (2015).

11.Iossifov, I., Ronemus, M., Levy, D., Wang, Z., Hakker, I., Rosenbaum, J., Yamrom, B., Lee, Y.H., Narzisi, G., Leotta, A., Kendall, J., Grabowska, E., Ma, B., Marks, S., Rodgers, L., Stepansky, A., Troge, J., Andrews, P., Bekritsky, M., Pradhan, K., Ghiban, E., Kramer, M., Parla, J., Demeter, R., Fulton, L.L., Fulton, R.S., Magrini, V.J., Ye, K., Darnell, J.C., Darnell, R.B., Mardis, E.R., Wilson, R.K., Schatz, M.C., McCombie, W.R., & Wigler, M. De novo gene disruptions in children on the autistic spectrum. *Neuron* **74,** 285-299 (2012).

12. Konopka, G., Wexler, E., Rosen, E., Mukamel, Z., Osborn, G.E., Chen, L., Lu, D., Gao, F., Gao, K., Lowe, J.K., & Geschwind, D.H. Modeling the functional genomics of autism using human neurons. *Mol Psychiatry* **17**, 202-214 (2012).

13. Lim, E.T., Raychaudhuri, S., Sanders, S.J., Stevens, C., Sabo, A., MacArthur, D.G., Neale, B.M., Kirby, A., Ruderfer DM, Fromer M, Lek M, Liu L, Flannick J, Ripke S, Nagaswamy U, Muzny, D., Reid, J.G., Hawes A, Newsham, I., Wu Y, Lewis L, Dinh H, Gross S, Wang LS, Lin CF, Valladares, O., Gabriel, S.B., dePristo, M., Altshuler, D.M., Purcell SM; NHLBI Exome Sequencing Project, State MW, Boerwinkle, E., Buxbaum, J.D., Cook, E.H., Gibbs, R.A., Schellenberg, G.D., Sutcliffe, J.S., Devlin, B., Roeder, K., & Daly MJ. Rare complete knockouts in humans: population distribution and significant role in autism spectrum disorders. *Neuron* **77,** 235-242 (2013).

14. Liu, Y., Du, Y., Liu, W., Yang, C., Liu, Y., Wang, H., & Gong, X. Lack of association between NLGN3, NLGN4, SHANK2 and SHANK3 gene variants and autism spectrum disorder in a Chinese population. *PLoS One* **8**, e56639 (2013).

15. Nava, C., Lamari, F., Héron, D., Mignot, C., Rastetter, A., Keren, B., Cohen, D., Faudet, A., Bouteiller, D., Gilleron, M., Jacquette, A., Whalen, S., Afenjar, A., Périsse, D., Laurent, C., Dupuits, C., Gautier, C., Gérard, M., Huguet G, Caillet S, Leheup B, Leboyer, M., Gillberg, C., Delorme, R., Bourgeron, T., Brice, A., & Depienne, C. Analysis of the chromosome X exome in patients with autism spectrum disorders identified novel candidate genes, including TMLHE. *Transl Psychiatry* **2**, e179 (2012).

16. Neale, B.M., Kou, Y., Liu, L., Ma'ayan, A., Samocha, K.E., Sabo, A., Lin, C.F., Stevens, C., Wang LS, Makarov V, Polak P, Yoon, S., Maguire J, Crawford EL, Campbell NG, Geller ET, Valladares, O., Schafer C, Liu, H., Zhao, T., Cai, G., Lihm J, Dannenfelser, R., Jabado O, Peralta Z, Nagaswamy, U., Muzny, D., Reid, J.G., Newsham I, Wu Y, Lewis L, Han Y, Voight BF, Lim E, Rossin E, Kirby A, Flannick J, Fromer M, Shakir K, Fennell T, Garimella K, Banks E, Poplin R, Gabriel S, DePristo M, Wimbish JR, Boone BE, Levy SE, Betancur C, Sunyaev ,S., Boerwinkle, E., Buxbaum, J.D., Cook, E.H. Jr, Devlin, B., Gibbs, R.A., Roeder, K., Schellenberg, G.D., Sutcliffe, J.S., & Daly, M.J. Patterns and rates of exonic de novo mutations in autism spectrum disorders. *Nature* **485**, 242-245 (2012).

17. Noh, H.J., Ponting, C.P., Boulding, H.C., Meader, S., Betancur, C., Buxbaum, J.D., Pinto, D., Marshall, C.R., Lionel, A.C., Scherer, S.W., & Webber C. Network topologies and convergent aetiologies arising from deletions and duplications observed in individuals with autism. *PLoS Genet* **9**, e1003523 (2013).

18. O'Roak, B.J., Vives, L., Fu, W., Egertson, J.D., Stanaway, I.B., Phelps, I.G., Carvill, G., Kumar, A., Lee, C., Ankenman, K., Munson, J., Hiatt JB, Turner EH, Levy R, O'Day DR, Krumm N, Coe BP, Martin BK, Borenstein E, Nickerson, D.A., Mefford, H.C., Doherty, D., Akey, J.M., Bernier R, Eichler EE, & Shendure J. Multiplex targeted sequencing identifies recurrently mutated genes in autism spectrum disorders. *Science* **338**, 1619-1622 (2012).

19. Sanders, S.J., Murtha, M.T., Gupta, A.R., Murdoch, J.D., Raubeson, M.J., Willsey, A.J., Ercan-Sencicek, A.G., DiLullo, N.M., Parikshak, N.N., Stein JL, Walker MF, Ober GT, Teran NA, Song, Y., El-Fishawy P, Murtha, R.C., Choi, M., Overton JD, Bjornson RD, Carriero, N.J., Meyer, K.A., Bilguvar, K., Mane SM, Sestan N, Lifton RP, Günel M, Roeder K, Geschwind DH, Devlin, B., & State, M.W. De novo mutations revealed by whole-exome sequencing are strongly associated with autism. *Nature* **485**, 237-241 (2012).

20. Shi, L., Zhang, X., Golhar, R., Otieno, F.G., He, M., Hou, C., Kim, C., Keating, B., Lyonm G.J., Wang, K., & Hakonarson, H. Whole-genome sequencing in an autism multiplex family. *Mol Autism* **4**, 8 (2013).

21. Tilot, A.K., Bebek, G., Niazi, F., Altemus, J.B., Romigh, T., Frazier, T.W., & Eng, C. Neural transcriptome of constitutional Pten dysfunction in mice and its relevance to human idiopathic autism spectrum disorder. *Mol Psychiatry* **21,** 118-125 (2016)

22. Toma, C., Torrico, B., Hervas, A., Valdes-Mas, R., Tristan-Noguero, A., Padillo, V., Maristany, M., Salgado, M., Arenas, C., Puente, X.S., Bayes, M., & Cormand, B. Exome sequencing in multiplex autism families suggests a major role for heterozygous truncating mutations. *Mol Psychiatry* **19**, 784-90 (2014).

23. Wang, J., Tao, Y., Song, F., Sun, Y., Ott, J., & Saffen, D. Common Regulatory Variants of CYFIP1 Contribute to Susceptibility for Autism Spectrum Disorder (ASD) and Classical Autism. *Ann Hum Genet* **24,** 329-340 (2015).

24.Yu, T.W., Chahrour, M.H., Coulter, M.E., Jiralerspong, S., Okamura-Ikeda, K., Ataman, B, Schmitz-Abe K, Harmin, DA, Adli, M, Malik, AN, D'Gama, AM, Lim, ET, Sanders, SJ, Mochida, GH, Partlow, JN, Sunu, CM, Felie, JM, Rodriguez J, Nasir, RH, Ware J, Joseph, RM, Hill, RS, Kwan, BY, Al-Saffar, M, Mukaddes, NM, Hashmi, A, Balkhy, S, Gascon, GG, Hisama, FM, LeClair, E, Poduri A, Oner O, Al-Saad S, Al-Awadi SA, Bastaki L, Ben-Omran T, Teebi AS, Al-Gazali L, Eapen V, Stevens CR, Rappaport L, Gabriel, SB, Markianos K, State, MW, Greenberg, ME, Taniguchi H, Braverman NE, Morrow EM, & Walsh, C.A. Using whole-exome sequencing to identify inherited causes of autism. *Neuron* **77**, 259-273 (2013).

25. Yuen RK, Thiruvahindrapuram B, Merico D, Walker S, Tammimies K, Hoang N, Chrysler C4, Nalpathamkalam T, Pellecchia G, Liu Y, Gazzellone MJ, D'Abate L, Deneault E, Howe JL, Liu RS, Thompson A, Zarrei M, Uddin M, Marshall CR, Ring RH, Zwaigenbaum L, Ray PN, Weksberg R, Carter MT, Fernandez BA, Roberts W, Szatmari P, Scherer SW. Whole-genome sequencing of quartet families with autism spectrum disorder. *Nat Med* **21**, 185-191 (2015).

26. SFARI (2014-2015)
