# Supplementary material for: Altered steady state and activity-dependent de novo protein expression in fragile X syndrome
Source: Nat Commun. 2019 Apr 12;10:1710. doi: 10.1038/s41467-019-09553-8 (PMC6461708; doi:10.1038/s41467-019-09553-8)
Supplement: Supplementary file 2 — Description of Additional Supplementary Files [file 41467_2019_9553_MOESM2_ESM.pdf]

Description of Additional Supplementary Files

File Name: Supplementary Data 1
Description: **WT vs KO steady state results.** Forward indicates KO/WT (FXS) and reverse indicates WT/FXS. There were three biological replicates performed. Consistent candidates were selected based on consistency across runs (2/2 or 3/3) with a Perseus Significance of P<0.30.

File Name: Supplementary Data 2
Description: **Top 20 DAVID GO clusters with P Value and FDR.** All proteins that met C-score criteria were compared with our hippocampal proteome database in DAVID 6.8**.**

File Name: Supplementary Data 3
Description: **Complete mass spectrometry results from DHPG studies.** Comparison of FXS, WT, and differential analysis of DHPG targets.

File Name: Supplementary Data 4
Description: **Top 20 DAVID GO table of top Functional Clusters for KO + DHPG consistently changed proteins.** Proteins consistently changed in KO + DHPG were compared to our hippocampal slice proteome database in DAVID 6.8.

File Name: Supplementary Data 5
Description: **Comparison with other published FXS and ASD databases.** Comparison was made via databases derived from the literature (details in 'database list' tab).'Partial' overall is indicated if the majority of the protein id matched but there were slight is from differences. Individual overlaps with steady state and DHPG lists are indicated by the tab name.
